# Peptide aptamer targeting Aβ–PrP–Fyn axis reduces Alzheimer’s disease pathologies in 5XFAD transgenic mouse model

**DOI:** 10.1007/s00018-023-04785-w

**Published:** 2023-05-07

**Authors:** Tahir Ali, Antonia N. Klein, Alex Vu, Maria I. Arifin, Samia Hannaoui, Sabine Gilch

**Affiliations:** 1 Calgary Prion Research Unit, Department of Comparative Biology & Experimental Medicine, Faculty of Veterinary Medicine, University of Calgary, 3330 Hospital Drive NW, Calgary, AB T2N 4Z6 Canada; 2 Cumming School of Medicine, Hotchkiss Brain Institute, University of Calgary, Calgary, AB Canada

**Keywords:** Alzheimer’s disease, Alzheimer’s disease treatment, Prion protein PrP, Peptide aptamer, Aβ oligomer, Aβ–PrP interaction, Fyn kinase, Gliosis, Apoptotic neurodegeneration, Cognitive function

## Abstract

Currently, no effective therapeutics exist for the treatment of incurable neurodegenerative diseases such as Alzheimer’s disease (AD). The cellular prion protein (PrP^C^) acts as a high-affinity receptor for amyloid beta oligomers (AβO), a main neurotoxic species mediating AD pathology. The interaction of AβO with PrP^C^ subsequently activates Fyn tyrosine kinase and neuroinflammation. Herein, we used our previously developed peptide aptamer 8 (PA8) binding to PrP^C^ as a therapeutic to target the AβO–PrP–Fyn axis and prevent its associated pathologies. Our in vitro results indicated that PA8 prevents the binding of AβO with PrP^C^ and reduces AβO-induced neurotoxicity in mouse neuroblastoma N2a cells and primary hippocampal neurons. Next, we performed in vivo experiments using the transgenic 5XFAD mouse model of AD. The 5XFAD mice were treated with PA8 and its scaffold protein thioredoxin A (Trx) at a 14.4 µg/day dosage for 12 weeks by intraventricular infusion through Alzet^®^ osmotic pumps. We observed that treatment with PA8 improves learning and memory functions of 5XFAD mice as compared to Trx-treated 5XFAD mice. We found that PA8 treatment significantly reduces AβO levels and Aβ plaques in the brain tissue of 5XFAD mice. Interestingly, PA8 significantly reduces AβO–PrP interaction and its downstream signaling such as phosphorylation of Fyn kinase, reactive gliosis as well as apoptotic neurodegeneration in the 5XFAD mice compared to Trx-treated 5XFAD mice. Collectively, our results demonstrate that treatment with PA8 targeting the AβO–PrP–Fyn axis is a promising and novel approach to prevent and treat AD.

**Supplementary Information:**

The online version contains supplementary material available at 10.1007/s00018-023-04785-w.

## Introduction

Alzheimer’s disease (AD) is a devastating, chronic, and incurable neurodegenerative disorder. AD is the major cause of dementia, which accounts for 60–70% of all dementia cases. Globally, there are over 50 million AD patients affected and this number is expected to increase to more than 150 million by 2050. Besides the health problem for patients and their families, AD also represents a socioeconomic burden, with estimated global costs of US$1 trillion annually, which will be doubled by 2030 [1]. Neuropathologically, AD is characterized by the presence of two main hallmarks, senile plaques composed of amyloid beta (Aβ) aggregates and neurofibrillary tangles (NFTs), containing hyperphosphorylated tau proteins within the brains of AD patients [2, 3]. However, since 1992, several studies reported and validated that Aβ aggregation is a primary event in the AD pathogenesis [4–7]. The Aβ peptide is produced by the proteolytic cleavage of the amyloid precursor protein (APP) via enzymatic action of β-secretase and γ-secretase. The Aβ peptides assemble from monomers into various multimeric species from small oligomers to fibrils. Most importantly, oligomeric species of Aβ peptide (AβO) are considered the primary neurotoxic agent to instigating synaptic deficit, memory impairments, and neurodegeneration [5–10]. However, yet the exact underlying cellular and molecular mechanism by which AβO triggers these pathological cascades causing brain degeneration remains unclear. Thus, it is required to use compounds which directly target AβO and its downstream mediators.

Since a decade and a half, mounting studies were carried out to identify cellular AβO receptors. Among the potentially crucial molecules, the cellular prion protein (PrP^C^) was identified as a high-affinity neuronal receptor of AβO [11]. Since then, the PrP^C^–AβO interaction and its effects have been extensively studied and validated in in vitro and in vivo models of AD [12–14]. PrP^C^ is an evolutionarily highly conserved glycosylphosphatidylinositol-anchor membrane protein that is present in mammals. PrP^C^ is a glycoprotein predominantly expressed in the neuronal cells of the brain and primarily implicated in prion diseases, neurodegenerative diseases caused by the accumulation of a misfolded and infectious isoform of PrP^C^, which is known as scrapie prion protein (PrP^Sc^) [15]. Despite its role as a receptor for AβO and substrate for PrP^Sc^ propagation, PrP^C^ is a copper binding protein, has a neuroprotective function and is involved in the formation and maintenance of synapses, and transmembrane signaling [16–19]. It was extensively reported that the AβO–PrP complex triggers various downstream kinases, including the activation of Fyn kinase, a non-receptor tyrosine kinase. Activated Fyn further triggers the downstream mediators and pathological events of the AβO-PrP axis [12–14, 19]. Thus, these evidences suggest that the AβO-PrP interaction and its associated downstream signaling escalate neurotoxicity and neuronal death in AD. Hence, targeting the interaction of AβO-PrP is a valuable approach to prevent toxic signaling and to treat AD.

Our group previously developed peptide aptamers binding to PrP^C^ as a group of compounds that can be used as a potential therapeutic tool to prevent protein misfolding neurodegenerative diseases such as prion diseases. We selected peptide aptamer 8 (PA8) from a combinatorial 16mer peptide library presented by the scaffold protein thioredoxin A (Trx) and expressed it in *Escherichia coli* (*E. coli*). Binding of PA8 to PrP^C^ efficiently interferes with PrP^Sc^ propagation and prevents prion infection of neuronal cells. Interestingly, our group showed that PA8 is binding to amino acids 100–120 PrP, which partly overlaps with the major binding site of AβO. In addition, PA8 binding to PrP enhances α-cleavage of PrP^C^. The α-cleavage releases a soluble, neuroprotective fragment N1 of PrP^C^ likely adding to a protective role in prion disease [20–24]. Peptide aptamers are small combinatorial peptides (5–20 amino acid) presented by a scaffold protein that can be screened and selected to bind to specific target molecules with high specificity and affinity. Insertion into the scaffold protein, e.g., Trx, stabilizes their conformation, conferring increased binding affinity compared to native peptides. They have a lower molecular weight and can be expressed in *E. coli* with high yield and low cost. Therefore, they are advantageous compared to therapeutic antibodies [25–27]. Currently, numerous comprehensive reviews highlight the importance of peptide aptamers, which have gained increasing attention of researchers and clinicians worldwide for therapeutic, diagnostics, and drug delivery purposes for various chronic and incurable diseases [27–32].

In this study, we aim to determine the potential protective and therapeutic effect of PA8 in in vitro and in vivo AD models. We found that PA8 prevents the binding of AβO to PrP and reduces toxicity of AβO in a neuronal cell line and primary hippocampal neurons. Most importantly, we observed that chronic treatment with PA8 significantly improves memory function of 5XFAD mice, a mouse model of AD, expressing human APP and presenilin-1 (PSEN1) transgenes harboring in total five mutations associated with familial AD. Our biochemical and histopathological assessment also showed that PA8 reduces AβO levels, Aβ plaques accumulation, AβO–PrP signaling and its downstream marker, p-Fyn, as well as activated gliosis and neurodegeneration in the brains of 5XFAD mice.

## Materials and methods

### Animals

Female 5XFAD (B6SJL-Tg (APPSwFlLon, PSEN1*M146 L*L286 V)6799Vas/Mmjax) transgenic mice were purchased from Jackson Laboratory, USA. The mice were kept at 12-h (hr)/12-h light/dark cycle and a maintained temperature at 23 °C, in an environment with 60 ± 10% humidity. The mice were allowed to access food and water ad libitum. All experiments related to mice in this study were approved by the University of Calgary Health Sciences Animal Care Committee (AC18-0030) according to the guidelines issued by the Canadian Council for Animal Care and ARRIVE guidelines.

### Expression and purification of recombinant PA8

The recombinant PA8 expression and purification was performed as described previously [20, 22]. Briefly, the sequences encoding Trx, used as a scaffold protein and PA8 were cloned into the *E. coli* expression vector pQE30 (Qiagen) as fusion to a N-terminal poly-histidine (6His) tag and then co-transformed with pREP4 into BL21-Gold (DE3) pLysS chemically competent cells (Agilent Technologies). Following dialysis, protein concentrations were determined by BCA assay (Thermo Fisher Scientific, USA) and concentrated to 3 mg/ml by size exclusion chromatography (Amicon centrifugal filter units, EDM Millipore). Protein purity was assessed by SDS-PAGE (12.5%) followed by Coomassie blue staining (Fig. S1A). To test the stability of PA8, we kept them at 37 °C for 6 weeks and 12 weeks, respectively. The stability was assessed for fresh PA8 as well as correspondingly after 6- and 12-weeks incubation at 37 °C using SDS-PAGE (12.5%) followed by Coomassie blue staining (Fig. S1B–D). Following purification and stability, we proceeded with the PA8 for both in vitro and in vivo studies.

### Animal treatment

Following acclimatization for 1 week, we randomly split 6-week-old female 5XFAD mice into two groups (Fig S2A) for treatment with PA8 (10 mice/group) and Trx (5 mice/group), respectively. Trx-treated mice served as a control group. The administration of PA8 and Trx was performed using intraventricular infusion through Alzet^®^ osmotic pumps according to the well-established protocol [33, 34]. We used the Alzet pump #2006 with a reservoir volume of 200 μl, which releases 0.15 μl/h. The concentration of purified PA8 and trxA was 4 mg/ml, resulting in a dosage of 14.4 μg/24 h. After 6 weeks, we replaced the Alzet® osmotic pumps very carefully by new ones according to the protocols and administered fresh PA8 and Trx at 14.4 µg/day dosage for another 6 weeks. Overall, the mice received PA8 and Trx at 14.4 µg/day dosage for 12 weeks (Fig. S2B). Three animals of the PA8 treatment group had to be euthanized due to complications following the second surgery and before the experimental end point and behavioral experiments. Issues included difficult wound healing and displacement of the osmotic pump tubing.

### Behavioral experiments

After completion of treatment, we carried out contextual fear conditioning and open field tests to assess the memory functions and anxiety type behavior of PA8- (n = 7 mice) and Trx- (n = 5 mice) treated 5XFAD mice.

### Contextual fear conditioning test

Fear conditioning is conducted in a chamber with plastic walls and a metal mesh floor. The internal dimensions of the chamber are approximately 17 cm × 17 cm × 25 cm. Before starting the training and testing, mice were acclimatized for 1–2 h in the behavioral rooms. After acclimatization, on day 1, mice were placed into the conditioning chamber and habituated to their surrounding for at least 2 min. Following habituation, the mice received three pairings of tone/light signals (20 s, 80 dB; or user specific setting) and a co-terminating electric shock (1 s, 0.5 mA). The inter-trial interval between each of the pairings was 2 min. Once the trial was complete, the mice were returned to their home cage. The chamber was cleaned with 70% ethanol after each mouse. On the final testing day, each animal was then placed in the same chamber for approximately 6 min (testing). The tone/light in the absence of shocks was presented twice 20 s, 120 s, and 260 s after the animal was placed into the chamber. During this testing period, the behavior of the mouse was recorded by a digital video camera directly mounted above the conditioning chamber. The amount of time spent freezing is quantified and defined by the complete absence of motion. Fear conditioning was conducted with the ANY-maze (Stoelting Co. UK). Freezing detection is automatically quantified by the software with the default freezing detection settings.

### Open field test

The spontaneous locomotory and exploratory as well as anxiety behaviors were evaluated using an open field container (60 × 60 cm), with a 5 × 5 grid drawn on the surface of the floor (each individual square is 12 × 12 cm). The open field box was divided into 16 equally sized squares, and the movements of the mice were recorded using a video tracking software (ANY-maze Stoelting Co., UK). The mice were acclimatized to the behavioral room for 2–3 h prior to the experiment. After acclimatization, each mouse was individually placed in the same position of the corner. Each mouse was allowed to explore their environment for 10 min. After each mouse, the box was cleaned with 70% ethanol and completely dried to avoid odor of previous mouse and their feces and urine. The experiments were carried out in a separate calm room to minimize distractions and accidentally cause freezing behavior. The total distance traveled by each mouse and the time spent in the central area were evaluated. The distance is presented in centimeters, while the time is presented in seconds.

### Preparation of brain homogenates and immunoblotting

Brain hemisphere homogenates were prepared according to our previously published protocol [35]. Briefly, one brain hemisphere was homogenized in 0.1 M phosphate buffered saline (PBS) (10% w/v) using a gentle MACS™ Dissociator for 2 min at room temperature, followed by centrifugation at 2,000 g for 1 min. The homogenates were aliquoted and stored at − 80 ℃ until further processing. For immunoblotting, 10% brain homogenates were mixed with equal volume of cold protein extraction buffer according to manufacture protocol (PROP-PREP™, Catalogue number: 17081, iNtron Biotechnology, USA). Protein samples were prepared in SDS sample buffer and processed on 12.5–18% SDS-PAGE. Electroblotting was done using Amersham Hybond P 0.45 PVDF membranes (Amersham, USA). Membranes were incubated with primary antibodies (Table 1) at 4 ℃ overnight. The membranes were washed three times each for 5 min. Following washing, the membranes were incubated in secondary antibodies according to the source of primary antibodies at room temperature at least for 1 h. After secondary antibody incubation, membranes were washed three times each for 5 min and then analyzed using Luminata Western Chemiluminescent HRP Substrates (Millipore, USA). The densitometric analysis of immunoblots was performed using ImageJ.

**Table 1:** List of primary antibodies and their detailed information

Primary antibodies	Host	Applications	Manufacturers/providers	Catalog/reference number	Concentrations
Aβ (6E10)	Mouse	WB/IF	Biolegend, USA	803,001	1:1000/1:100
PrP	Rabbit	IF	Invitrogen, ThermoFisher Scientific, USA	CD230	1:100
p-Tyr 416 (p-Fyn)	Rabbit	WB/IF	=	PA597368	1:1000/1:100
Caspase 3	Rabbit	WB	Cell signaling, USA	9662S	1:1000
p-Tyr 416 (p-Fyn)	Rabbit	WB/IF	=	2101S	1:1000/1:100
Fyn (15)	Mouse	WB	SantaCruz Biotechnology, USA	SC-434	1:1000
GFAP	Mouse	WB/IF	=	SC: 33,673	1:40,000/1000
Iba-1	Mouse	WB	=	SC: 32,725	1:1000
p-JNK	Mouse	WB	=	SC: 6254	1:1000
JNK	Mouse	WB	=	SC: 6254	1:1000
Synaptophysin (Synap)	Mouse	WB	=	SC: 17,756	1:1000
Cytochrome C (Cyt. C)	Mouse	WB	Novus Biologicals, LLC, USA	NB100-56,503	1:1000
Iba-1	Goat	IF	=	NB100-1028	1:25
GFAP	Rabbit	IF	=	BN300-141	1:1500
m4H11	Mouse	WB	[22]	[22]	1:1000
β-Actin	Mouse	WB	Sigma Aldrich, USA	A5441	1:40,000

### Collection of mouse brain sections for morphological analyses

On completion of behavioral experiments, mice were deeply anesthetized with isoflurane and then transcardially perfused with 0.1 M PBS and 4% paraformaldehyde. The brain hemispheres were post-fixed in 4% paraformaldehyde for 72 h and brain tissues were cryoprotected with 20% sucrose for 72 h in 0.1 M PBS at 4 ℃ for 3–5 days until they completely sank. Brains were frozen in optimum cutting temperature compound (A.O, USA), and 12 μm coronal sections were collected using a CM 3050C cryostat (Leica, Germany). Brain tissue sections were thaw-mounted on commercially available ProbeOn Plus charged slides (Fisher, USA).

### Immunofluorescence staining, confocal microscopy, and stereological analyses

Single and double immunofluorescence staining was performed as described previously with some modification [35, 36]. Briefly, gelatin-coated slides containing brain tissues were dried 2–4 h at room temperature. After drying, the slides were washed twice for 5 min each in 0.01 M PBS. Following washing, the tissues were processed for antigen retrieval using 1X proteinase-k enzymatic step. The tissues were incubated for 10 min at room temperature. The slides were washed twice for 5 min each, followed by incubation for 1 h in blocking solution containing 2% serum and 0.1% Triton X-100 in 0.01 M PBS according to the source of primary antibodies. After blocking, the slides containing tissues were incubated overnight at 4 °C in the primary antibodies (Table 1). After primary antibody incubation, the sections were washed twice for 5 min each and incubated for at least for 1 h with donkey anti-mouse IgG H&L (FITC) (ab6816, Abcam, USA), rhodamine (TRITC) donkey anti-goat IgG (H + L) alexa fluor 488, goat anti-rabbit, or alexa fluor™ 555 goat anti-rabbit secondary antibodies (Jackson Immunoresearch, USA) (1:100). For double immunofluorescence, following incubation with the secondary antibodies, the slides were washed twice for 5 min each and then incubated overnight with the second primary antibodies. Following incubation with the second primary antibodies (Table 1), the sections were incubated with another secondary antibodies donkey anti-mouse IgG H&L (FITC) (ab6816, Abcam, USA), rhodamine (TRITC) donkey anti-goat IgG (H + L) alexa fluor 488, goat anti-rabbit, or alexa fluorTM 555 goat anti-rabbit secondary antibodies (Jackson Immunoresearch, USA) (1:100) for 1 h at room temperature. After overnight incubation, slides were washed twice for 5 min each and then coverslips were mounted with DAPI along with Dako fluorescent mounting medium (Molecular Probe, Eugene, OR). The immunofluorescence images were captured at same conditions for all images using a confocal laser scanning microscope (Zeiss LSM 700 confocal microscope). Several images per section (tissue) and more than 20 images per field of each area of the brain were captured from each respective group. Confocal images were converted to tagged image file format (TIF). The quantification of the immunofluorescence intensity in the same region of the brain areas in the TIF images for all groups was performed using ImageJ software. The background of TIF images was optimized according to the threshold intensity, and the immunofluorescence intensity was analyzed at specified threshold intensity for all groups at same conditions and was expressed as the relative integrated density between the groups.

### Preparation of AβO for in vitro experiments

The Aβ_1-42_ peptide (Sigma Chemical Co., St. Louis, MO, USA) was incubated in 100% hexafluoro isopropanol (HFIP) overnight at room temperature. After overnight incubation, HFIP was evaporated under vacuum and the dry Aβ was collected and stored at − 80 ℃. The peptides were reconstituted in DMSO (dimethyl sulfoxide) to collect a suspension of 100 mM, diluted with F12 medium (Gibco by life technologies, Grand Island, NY, USA) to 100 µM and incubated at room temperature for 16 h. The peptide solution was then centrifuged at 21,000 g at 4 ℃ for 15 min. The supernatant was collected as the AβO. To confirm the AβO species for every batch of preparation before the experiment, we performed immunoblotting using Tris-Tricin gels (12.5–16.5%).

### In vitro experiments for AβO binding with PrP

The murine neuroblastoma cell line N2a was purchased from ATCC (CCL-131), USA. N2a cells were cultured in Opti-MEM Glutamax medium (GIBCO, USA) with 10% fetal bovine serum (FBS), and penicillin/streptomycin at 37 °C in a 5% CO2 atmosphere. After the cells reached 70–80% confluence, they were pretreated with 10 μg/mL PA8 and Trx overnight. Afterwards, the cells were incubated with 1 μM FITC-AβO (pre-aggregated for 3 h) for 30 min on ice. Then cells were washed with 0.01 M PBS, fixed with 4% paraformaldehyde, and again washed with 0.01 M PBS. Slides were mounted with DAPI and Prolong Antifade Reagent (Molecular Probe, Eugene, OR, USA). Representative images were captured using FITC and DAPI filters with a laser confocal microscope (Zeiss LSM 700 confocal microscope, USA).

### Primary hippocampal neuron culture

C57BL/6 mice (Charles River, Saint Constant, Quebec, Canada) were used for preparing primary cultures of hippocampal neurons. Embryos were collected at gestational day 18. The embryos were dissected, and brains were collected, and hippocampal neuronal cells extracted and plated at a density of 1 × 10^5^ cells/mm^2^ on tissue culture wells pre-coated with 10 μg/ml poly-D-lysine. Cells were cultured in neuronal feed (Neurobasal/B-27 culture medium) without L-glutamine (Invitrogen cat. no. 21103–049) supplemented with 2% B-27 supplement (Invitrogen cat. no. 17504–044), 2 mM GlutaMAX (Invitrogen cat. no. 35050–061) 100 U/ml penicillin and 100 μg/ml streptomycin (Invitrogen cat. no. 10378–018). Cells were incubated at 37 °C in a humidified 5% CO_2_ atmosphere. Cells were treated with 10 μg/mL PA8 or Trx and 1 μM AβO and staurosporine (1 μM) as a positive control for apoptosis. After 24 h of incubation, the cells were washed with 0.01 M PBS, fixed with 4% paraformaldehyde, and processed for TUNEL (Terminal deoxynucleotidyl transferase dUTP nick end labeling) assay.

### MTT (3-[4,5-dimethylthiazol-2-yl]-2,5-diphenyltetrazolium bromide) assay

Colorimetric MTT assay was performed using the Cell Proliferation Kit I (Roche, Germany) to analyze the viability of N2a cells. N2a cells were cultured in 96-well plates (1 × 10^5^ cells/well) in 200 μl of Opti-MEM Glutamax medium (GIBCO, USA) with 10% FBS, and penicillin/streptomycin at 37 °C in a 5% CO2 atmosphere. After 72 h of incubation and 70–80% confluency, the N2a cells were treated with 10 μg/mL PA8 or Trx and 1 μM AβO and incubated for 24 h. Following the treatment, MTT (5 mg/ml in PBS) solution was added to the corresponding wells, and the plates were incubated for 4 h at 37 °C. DMSO was added to the wells, and the plates were agitated for 10 to 20 min on a shaker to dissolve formazan crystals. The absorbance was then measured at 550–570 nm (L1) and 620–650 nm (L2) using a scanning microplate reader. The L2 absorbance measures cell debris and well imperfections. According to the manufacture protocol, the absorbance (A = L1-L2) of each well was used to calculate the percentage of cell survival as × 100 absorbance of treated wells/absorbance of control wells.

### Proximity ligation assay (PLA) or Duolink® PLA technology

The PLA (Duolink® PLA technology) is an advanced and highly sensitive redout to use for detection of specific protein–protein interactions in situ (at distance less than 40 nm) at endogenous protein levels. PLA kit was bought from Sigma (Duolink^®^ in situ), and the assay protocol was based on the manufacturer's protocols and other published reports with slight modifications [37, 38]. For PLA, the slides containing brain tissues were processed similar to immunofluorescence staining until primary antibody incubation. Following antigen retrieval and blocking steps, the sections were incubated with rabbit anti-PrP (CD230, Invitrogen, Thermofisher, USA) and mouse anti-Aβ 6E10 primary antibodies (Table 1). Once incubation with primary antibodies was complete, the sections were rinsed with Buffer A, and later incubated with PLA probes Rabbit-plus and Mouse-minus diluted in Duolink® antibody diluent for 1 h at 37 °C. Upon completion, the sections were rewashed with Buffer A, followed by incubation with DNA ligation reagents for 30 min at 37 °C. After another wash with Buffer A, enzymatic amplification and PLA hybridization were performed by adding the corresponding reagents onto the sections for 100 min at 37 ℃. Section were then washed in a dark room with buffer B to avoid signal bleaching from light. After a quick wash with buffer B, the coverslips were mounted with DAPI along with Dako fluorescent mounting medium (Molecular Probe, Eugene, OR). The immunofluorescence images were captured at same conditions for all images using a confocal laser scanning microscope (Zeiss LSM 700 confocal microscope). Several images per section (tissue) and more than 20 images per field of each area of the brain were captured from each respective group. Confocal images were converted to TIF. The number of punctate counts per field was then plotted for each group. To confirm specificity, AβO or PrP was omitted in the negative controls and other steps were performed as described above.

### TUNEL assay

To assess apoptotic neurodegeneration, we used an in situ cell death (TUNEL) assay kit. The TUNEL assay was performed according to the manufacturer’s protocol (Roche, Cat. No. 7791-13-1) for both in vitro and in vivo samples. Following the TUNEL staining, the slides were mounted with DAPI and Prolong Antifade Reagent (Molecular Probe, Eugene, OR), and the images were captured using laser confocal microscope (Zeiss LSM 700 confocal microscope). Five images per section (tissue) were captured from each respective group. Confocal images were converted to TIF. The quantification of the immunofluorescence intensity in the same region of the brain areas (cortex and hippocampus (CA1)) in the TIF images for both groups was performed using ImageJ software. The background of TIF images was optimized according to the threshold intensity, and the immunofluorescence intensity was analyzed at specified threshold intensity for all groups at same conditions and was expressed as the relative integrated density between the groups. The same conditions without adding the TUNEL reaction mixture were used in the negative control.

### Data and statistical analyses

The immunoblotting and confocal results were analyzed using Image J software. For the confocal images, the bar graphs were produced from the integrated density of immunofluorescence intensity for immunoreactivity of each staining assay in image J. However, we divided all integrated density (arbitrary units) by their average numbers, which gave us the relative integrated density values. The two-tailed independent Student’s *t* test was used for statistical analyses and histograms were produced using GraphPad Prism software (GraphPad 8, Software, USA). Values are expressed as mean ± SEM. Significance = *p ≤ 0.05, **p ≤ 0.01, and ***p ≤ 0.001.

## Results

### PA8 reduces AβO-induced neurotoxicity

**Fig. 1:**
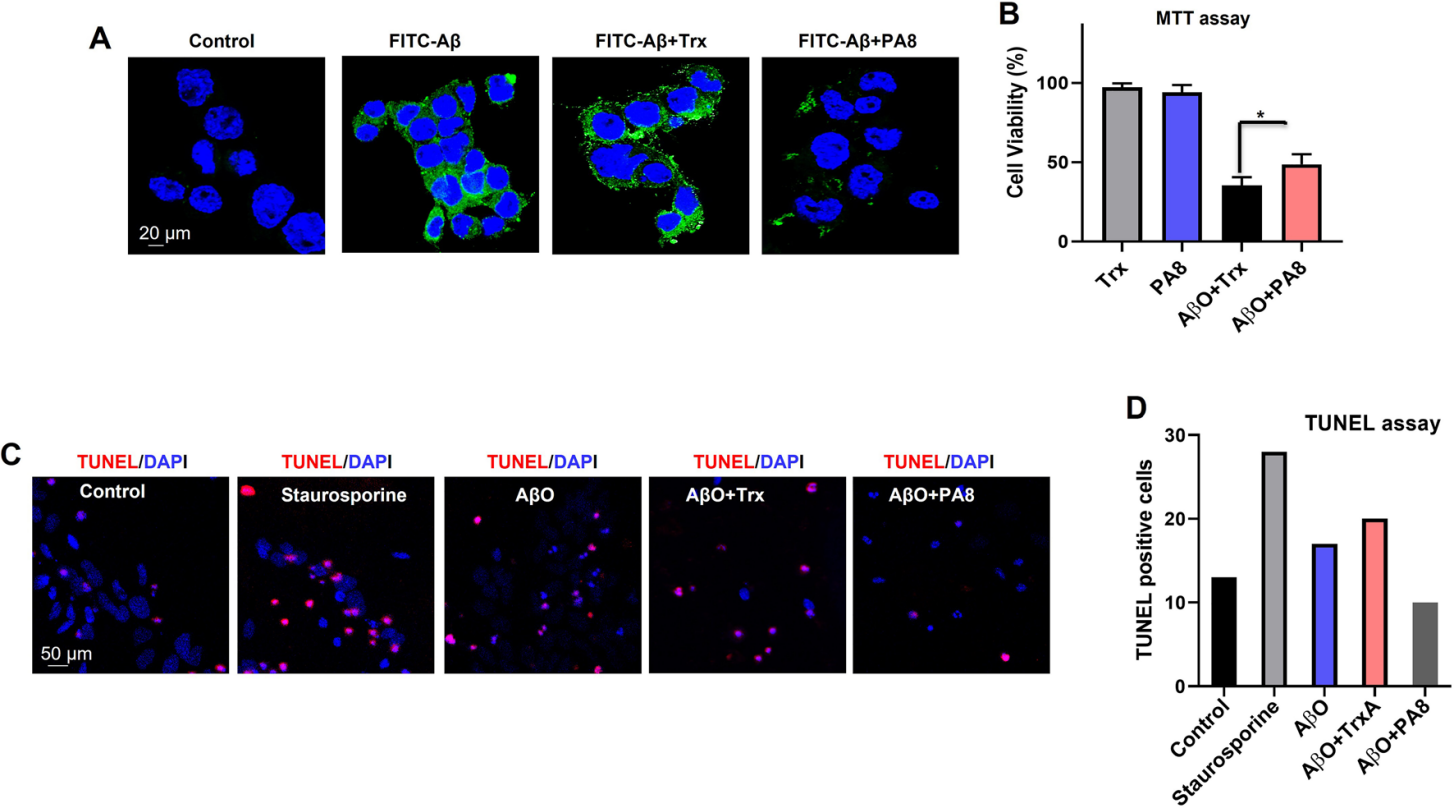
PA8 reduces AβO-induced neurotoxicity. **A** PA8 inhibits the binding of AβO with PrP. Representative confocal microscope images of N2a cells without any treatment as control, FITC-AβO, Trx-FITC-AβO and FITC-AβO-PA8 treated N2a cells. Magnification: 63X. Scale bar = 20 μm. **B** N2a cells were treated with AβO (1 µM) and PA8 or the Trx scaffold as a control (10 µg/ml), and after 24 h, MTT assay was performed. The data are expressed as the mean ± SEM of three independent MTT experiments = 3. Significance = *p < 0.05. **C** Confocal images of TUNEL-positive cells (red) and DAPI (blue) in primary hippocampal neurons. Staurosporine (1 μM) was used as a positive control and without any treatment as a negative control. **D** Histogram represents the average number of TUNEL-positive cells from five fields of view for each group. However, TUNEL assay was performed only one time, and therefore we did not perform statistical analysis

To determine whether PA8 inhibits the binding of AβO with PrP^C^, we performed confocal microscopy using N2a cells. The N2a cells were incubated with AβO-FITC (1 µg/ml) and the Trx scaffold as a control (10 µg/ml) as well as with Aβ-FITC (1 µg/ml) and PA8 (10 µg/ml). We found that PA8 (10 µg/ml) reduced the AβO immunoreactivity as compared to Trx (Fig. 1A). These results suggest that PA8 has the potential to prevent the binding of AβO to PrP^C^. Next, we performed MTT assays to assess the toxicity of AβO in the presence of Trx or PA8 in N2a cells. MTT results indicated that both Trx and PA8 did not affect the cell viability of N2a cells. We also found that AβO induced the same effect as of AβO + Trx on cell viability (Fig. S2) However, we observed a significant reduction of cell viability in N2a cells treated with AβO (1 µM) and Trx (10 µg/ml), while N2a cells treated with AβO (1 µM) and PA8 (10 µg/ml) had significantly improved cell viability as compared to Trx-treated cells (Fig. 1B; *p < 0.05). To confirm these results and assess the protective effect of PA8 in a more physiological system, we performed TUNEL assay in primary hippocampal neuron cultures. Notably, we observed fewer number of TUNEL-positive cells in the PA8 (10 µg/ml) as compared to AβO- (1 µM) and AβO + Trx-treated cells (Fig. 1C, D). Overall, these results in N2a cells and primary hippocampal neurons demonstrate that PA8 prevents the AβO interaction with PrP and reduces AβO-induced neurotoxicity.

### Administration of PA8 improved learning and memory functions of 5XFAD mice

**Fig. 2:**
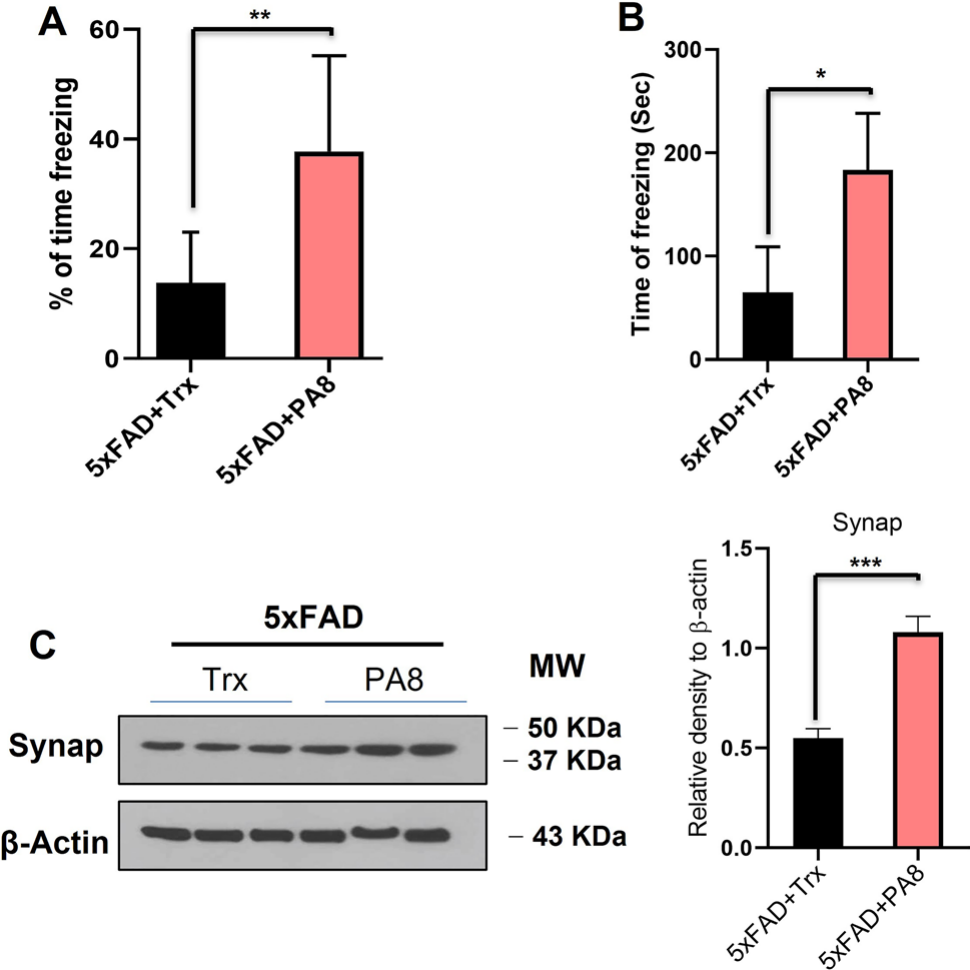
Administration of PA8 improves learning and memory functions of female 5XFAD mice. **A and B** In the contextual fear conditioning test, Trx-treated 5XFAD mice show significantly lower percentage of freezing and time of freezing than PA8-treated 5XFAD mice. The histograms show the means ± SEM for the mice (seven female mice for PA8 and five female mice for Trx treatment). Significance = *p < 0.05, **p < 0.01. **C** PA8 restored the level of pre-synaptic marker, synaptophysin. Immunoblotting and quantification of Synap levels in the three different brain homogenates of individual Trx- and PA8-treated 5XFAD mice. β-Actin was used as a loading control. The data are expressed as the means ± SEM for the representative proteins (*n* = 3 female mice/group) and the number of independent immunoblotting experiments = 3. Significance = ***p < 0.001

To evaluate the effect of PA8 treatment on AβO toxicity in vivo, we chose the 5XFAD model. These mice exhibit elevated intraneuronal aggregation of Aβ at 1.5 months of age, prior to Aβ neuritic plaque formation that begins at 2 months and is increased by 5 months. It is well-established that 5XFAD mice have memory deficits starting by 4 months of age and develop AD pathologies as compared to the non-transgenic mice on the same background [39–41]. Thus, to evaluate the effect of PA8 on memory functions, we used two groups of 5XFAD mice that underwent surgery for implantation of osmotic pumps for intracerebroventricular delivery of PA8 and Trx at an age of 6 weeks (Fig. S3A). One group of 5XFAD mice was treated with Trx, while the second group of 5XFAD mice was treated with PA8, both at a 14.4 µg/day dosage for 12 weeks (Fig. S3B). After completion of treatment, we assessed the memory functions of both groups of mice. We observed that in the fear conditioning test, on the testing day, the 5XFAD mice treated with PA8 had a remarkable increase in percentage of freezing and total time of freezing as compared with Trx-treated 5XFAD mice (Fig. 2A, B; **p < 0.01, *p < 0.05). In open field test, we did not observe any significant difference in total distance traveled and total time spent in the outer zones between PA8-treated and Trx-treated 5XFAD mice (Fig. 4SA, B), indicating that mice in both groups did not show any changes in anxiety or movement behavior.

It is well-established that altered synaptic proteins are correlated with memory deficits in AD. Therefore, we examined the effects of PA8 treatment on pre-synaptic markers such as Synaptophysin (Synap). Consistent with the result of behavioral experiments, we found that PA8-treated 5XFAD mice had a significantly higher Synap level in the brain homogenates as compared to Trx-treated 5XFAD mice (Fig. 2C; ***p < 0.001). Collectively, these behavioral and immunoblotting results of pre-synaptic Synap revealed that PA8 improved synaptic and memory functions, while there was no significant difference in anxiety and motor related behavior.

### Administration of PA8 ameliorates AβO levels, Aβ plaques, and its associated pathologies

**Fig. 3:**
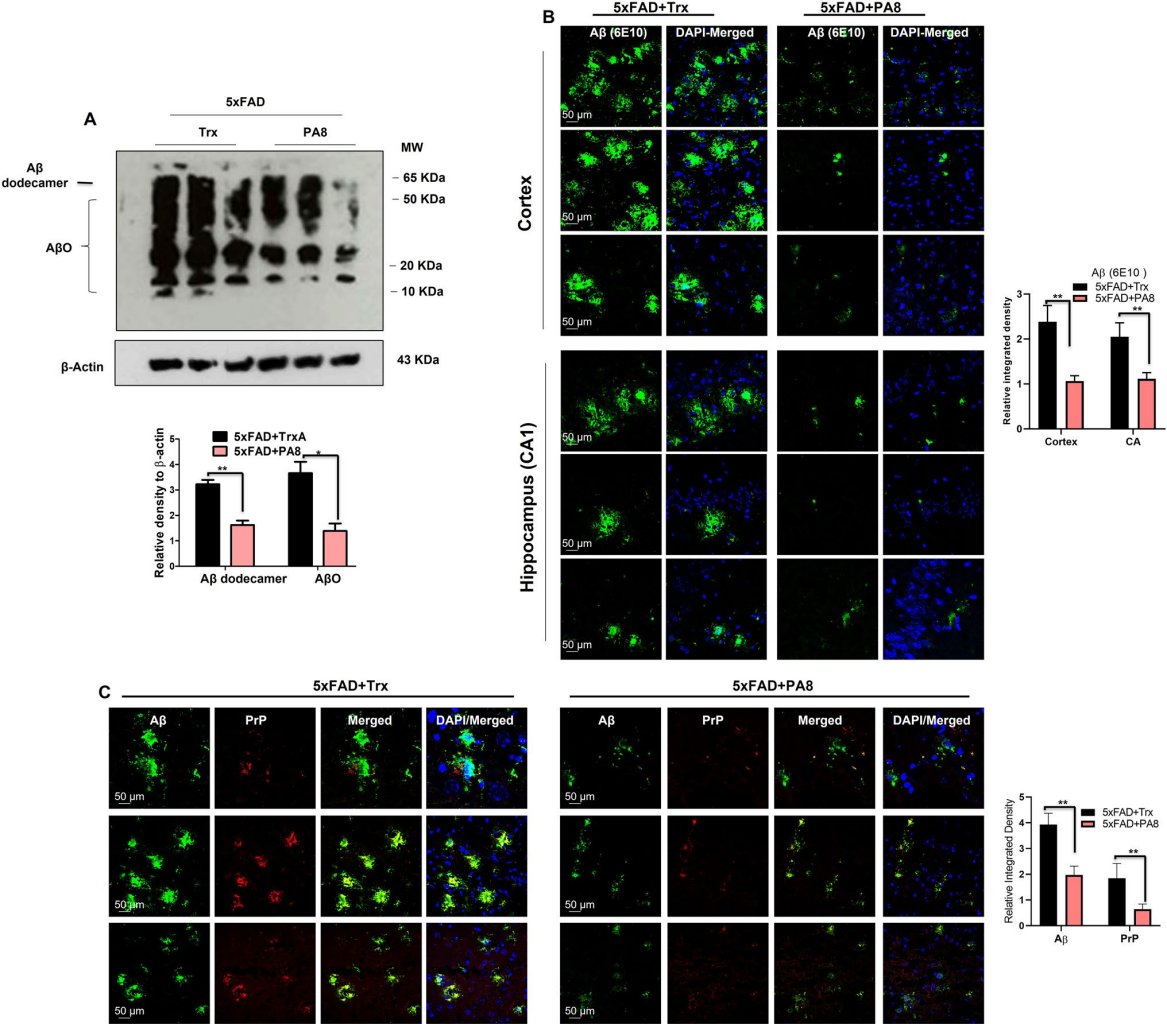
Administration of PA8 ameliorates AβO levels, Aβ plaques, and its associated pathologies. **A** Immunoblotting and quantification of Aβ dodecamer and AβO levels in three different brain homogenates of individual Trx- and PA8-treated 5XFAD mice. β-Actin was used as a loading control. The data are expressed as the means ± SEM for the representative proteins (*n* = 3 female mice/group) and the number of independent immunoblotting experiments = 3. Significance = *p < 0.05, **p < 0.01. **B** Confocal images of Aβ (6E10) (green) and DAPI (blue) in the cortices and hippocampi (CA1) of Trx- and PA8-treated 5XFAD mice. Data are expressed as the means ± SEM for *n* = 3 mice/group, and the number of independent confocal experiments = 3. **C** Confocal images of double immunofluorescence of Aβ (green) and PrP (red) in the cortices of Trx- and PA8-treated 5XFAD mice. Data are expressed as the means ± SEM for n = 3 female mice/group, and the number of independent confocal experiments = 3. Magnification: 63X. Scale bar = 50 μm. Significance = **p < 0.01

It is well-known that the 5XFAD model is ideal to assess the amount of AβO and Aβ plaques [39, 41]. Thus, we aimed to assess the effect of PA8 treatment (14.4 µg/day dosage for 12 weeks) on AβO and Aβ plaque burden in 5XFAD mice. We performed immunoblotting of brain homogenates of PA8 and Trx-treated 5XFAD mice. Using Aβ (6E10) antibody, we found that PA8 treatment significantly reduced the levels of AβO species and Aβ dodecamer as compared to Trx-treated 5XFAD mice (Fig. 3A; **p < 0.01, *p < 0.05).

**Fig. 4:**
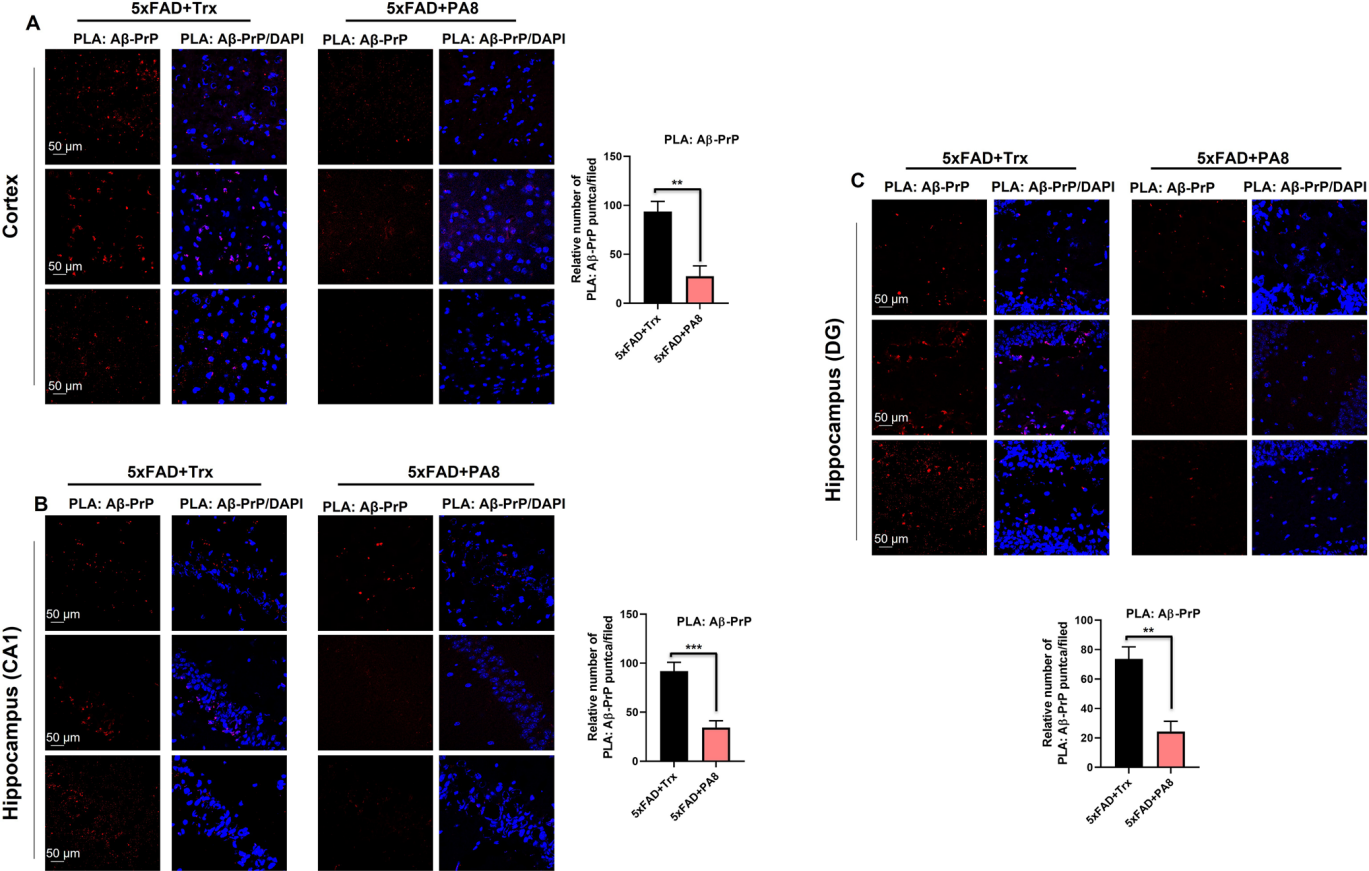
PA8 reduces the Aβ–PrP signaling. **A** Representative images of PLA signals and quantification for Aβ–PrP in the cortices of Trx- and PA8-treated 5XFAD mice. **B** and **C** Representative images of PLA signals and quantification for Aβ–PrP in the CA1 and DG regions of hippocampus of Trx- and PA8-treated 5XFAD mice. Data are expressed as the means ± SEM for *n* = 3 female mice/group, and the number of independent PLA confocal experiments = 3. Magnification: 63X. Scale bar = 50 μm. Significance = **p < 0.01; ***p < 0.001

Next, to confirm these results, we used confocal microscopy to analyze the brain tissue of PA8 and Trx-treated 5XFAD mice. We observed strong immunoreactivity for Aβ plaques in the cortical and hippocampus (CA1) of Trx-treated 5XFAD mice. However, we observed a lower number and less immunoreactivity for Aβ plaques in PA8-treated mice as compared to Trx-treated mice (Fig. 3B; **p < 0.01). Further, double immunofluorescence staining showed that PA8 treatment significantly decreased the relative immunoreactivity of Aβ (6E10) plaques and PrP in PA8-treated mice as compared to Trx-treated 5XFAD mice (Fig. 3C; **p < 0.01). Next, we performed PLA, to determine whether in addition to reducing Aβ (6E10) and PrP immunoreactivity, PA8 treatment interfered with the direct interaction between AβO and PrP. Consistent with the in vitro results (Fig. 1A) and the above results (Fig. 3A–C), PA8 treatment prevented the interaction of AβO with PrP, as represented by a significant decreased number of PLA positive puncta (number of puncta per field) in the cortical as well as CA1 and DG regions of the hippocampus compared to Trx-treated 5XFAD mice (Fig. 4A–C; **p < 0.01; ***p < 0.001). Moreover, to validate the PLA: AβO–PrP signals in the same brain region, we performed PLA without adding PrP antibody and showed that there were no PLA positive puncta signals (Fig. S5), confirming the specificity of PLA signals for the interaction of AβO–PrP. These results demonstrate that PA8 treatment reduces AβO pathologies and AβO–PrP interaction.

### PA8 reduces Fyn activation in 5XFAD mice

**Fig. 5:**
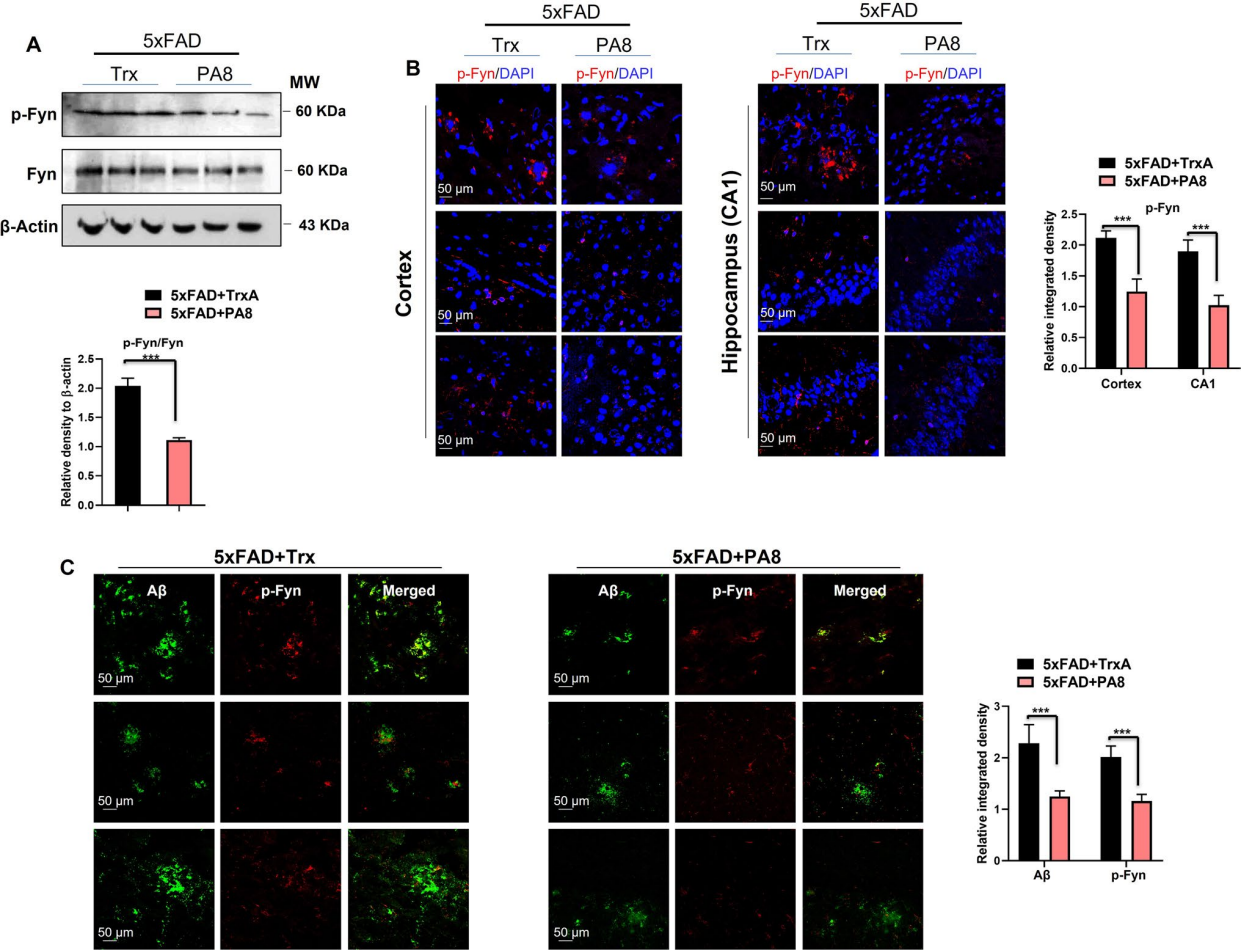
PA8 reduces the activated Fyn level in 5XFAD mice. **A** Immunoblotting of p-Fyn, total Fyn, and β-actin in the three different brain homogenates of individual Trx- and PA8-treated 5XFAD mice. We quantified the ratio of p-Fyn with total Fyn and then normalized with the β-actin. β-Actin was used as a loading control. The data are expressed as the means ± SEM for the representative proteins (*n* = 3 female mice/group) and the number of independent immunoblotting experiments = 3. Significance = ***p < 0.001. **B** Confocal images of p-Fyn (red) and DAPI (blue) in the cortex and CA1 region of hippocampus of Trx- and PA8-treated 5XFAD mice. Data are expressed as the means ± SEM for *n* = 3 female mice/group, and the number of independent confocal experiments = 3. **C** Confocal images of double immunofluorescence of Aβ (green) and p-Fyn (red) in the cortex region of brain of Trx- and PA8-treated 5XFAD mice. Data are expressed as the means ± SEM for n = 3 female mice/group, and the number of independent confocal experiments = 3. Magnification: 63X. Scale bar = 50 μm. Significance = ***p < 0.001

The interaction of AβO–PrP has been shown to trigger activation of intracellular Fyn in AD and other neurodegenerative diseases [16–18]. To determine the effect of PA8 on activation of Fyn, we performed immunoblotting using the p-Y416 Src family kinase (SFK) and total Fyn antibodies. We found that PA8 treatment decreased the p-Fyn levels, while it did not affect the total Fyn levels, indicating that PA8 treatment significantly reduced the Fyn activation as compared to Trx-treated 5XFAD mice (Fig. 5A; ***p < 0.001). Next, we performed confocal microscopy to corroborate the immunoblotting results and the effect of PA8 on p-Fyn. We observed that PA8 significantly reduced the p-Fyn immunofluorescence reactivity as compared to Trx-treated 5XFAD mice (Fig. 5B; ***p < 0.001). The double immunofluorescence results of Aβ and p-Fyn also showed that PA8 treatment significantly reduced relative immunoreactivity of Aβ and p-Fyn in 5XFAD mice, compared to Trx-treated 5XFAD mice. In summary, these findings indicate that PA8 reduces the amount of p-Fyn, likely associated with the attenuation of AβO–PrP interaction.

### PA8 reduces activated gliosis in 5XFAD mice

**Fig. 6:**
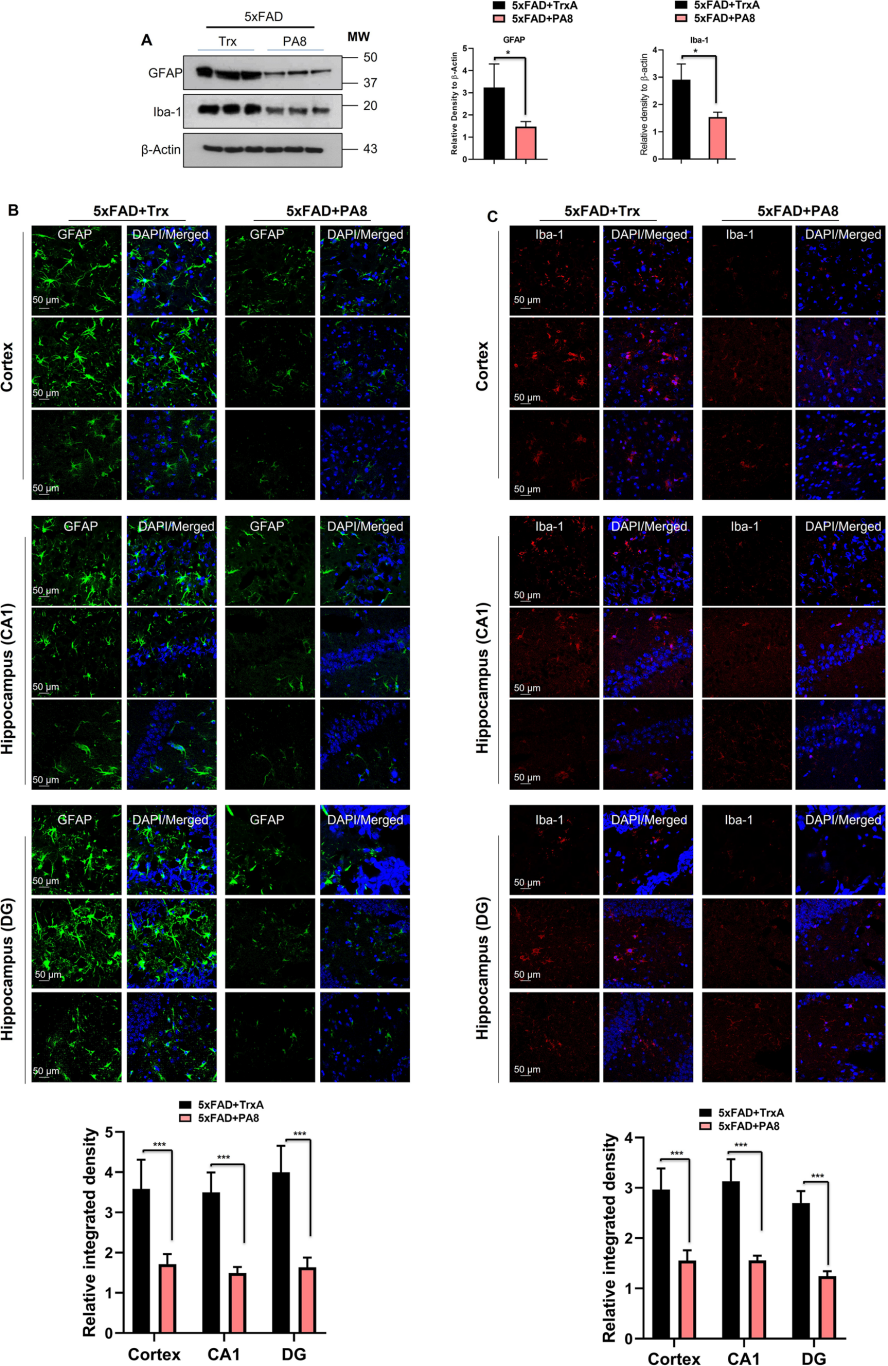
PA8 reduces the activated gliosis in 5XFAD mice. **A** Immunoblotting and quantification of GFAP, Iba-1, and β-actin in the three different brain homogenates of individual Trx- and PA8-treated 5XFAD mice. β-Actin was used as a loading control. The data are expressed as the means ± SEM for the representative proteins (*n* = 3 female mice/group) and the number of independent immunoblotting experiments = 3. Significance = *p < 0.05. **B** and **C** Confocal images of GFAP (green), Iba-1 (red), and DAPI (blue) in the cortex and CA1 and DG region of hippocampus of Trx- and PA8-treated 5XFAD mice. Data are expressed as the means ± SEM for *n* = 3 female mice/group, and the number of independent confocal experiments = 3. Magnification: 63X. Scale bar = 50 μm. Significance = ***p < 0.001

**Fig. 7:**
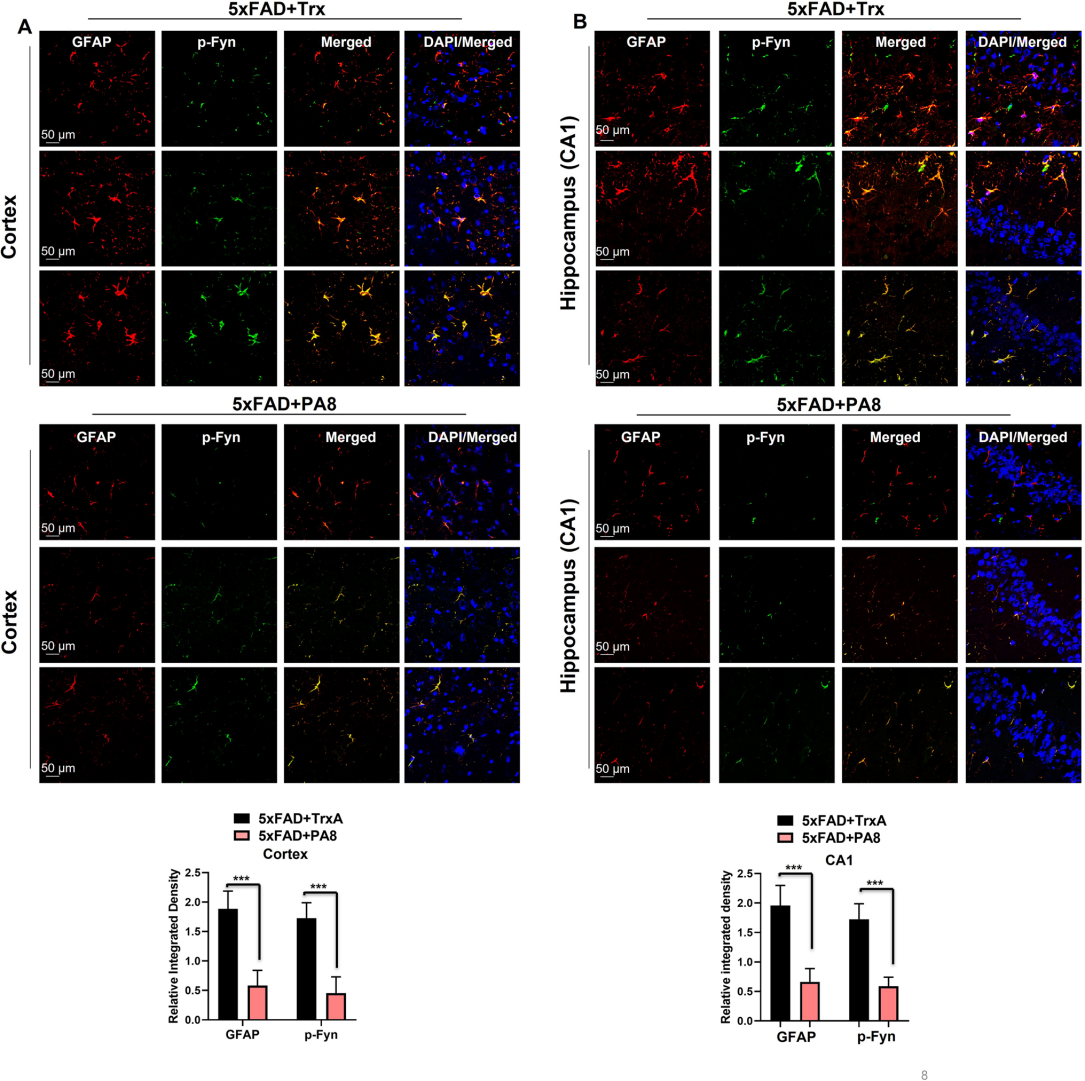
PA8 reduces activated p-Fyn in reactive astrocytes in 5XFAD mice. **A** Confocal images of double immunofluorescence of GFAP (red) and p-Fyn (green) in the cortex brain of Trx- and PA8-treated 5XFAD mice. **B** Confocal images of double immunofluorescence of GFAP (red) and p-Fyn (green) in the CA1 region of brain of Trx- and PA8-treated 5XFAD mice. Data are expressed as the means ± SEM for n = 3 female mice/group, and the number of independent confocal experiments = 3. Magnification: 63X. Scale bar = 50 μm. Significance = ***p < 0.001

Recently, several studies reported that activation of Fyn is associated with activated gliosis in protein misfolding neurodegenerative diseases and other brain disorder [42–44]. Therefore, following the observed significant effect of PA8 on reduction of p-Fyn, we aimed to determine the effect of PA8 on activated gliosis. For activated astrocytes and microglia, we used specific markers such as glial fibrillary acidic protein (GFAP) and ionized calcium binding adapter molecule 1 (Iba-1), respectively. Our immunoblotting results revealed that PA8 significantly reduced GFAP and Iba-1 levels as compared to Trx-treated 5XFAD mice (Fig. 6A; *p < 0.05). This was confirmed by confocal microscopy images showing that PA8 significantly decreased the GFAP and Iba-1 immunofluorescence reactivity in the cortical and hippocampal (CA1, DG) regions of the brain as comparted to Trx-treated 5XFAD mice (Fig. 6A, B; ***p < 0.001). Double immunofluorescence assays revealed activated astroglia and microglial cells around the plaques in the brains of Trx-treated mice, which was significantly reduced in the PA8-treated mice (Fig. S6A, B; **p < 0.01). Next, we also performed double immunofluorescence of p-Fyn and GFAP to examine whether the increased reactivity of p-Fyn is associated with reactive astrocytes. We found that PA8 remarkably alleviated the reactivity of p-Fyn and reactive astrocytes as compared to Trx-treated 5XFAD mice (Fig. 7A, B; Fig. S7 ***p < 0.001). Overall, these results indicate that PA8 reduces Aβ plaques-associated activated gliosis, which might be associated with the reduction of its downstream mediator, activated Fyn kinase.

### PA8 reduces stress kinase and apoptotic neurodegeneration-related signaling

**Fig. 8:**
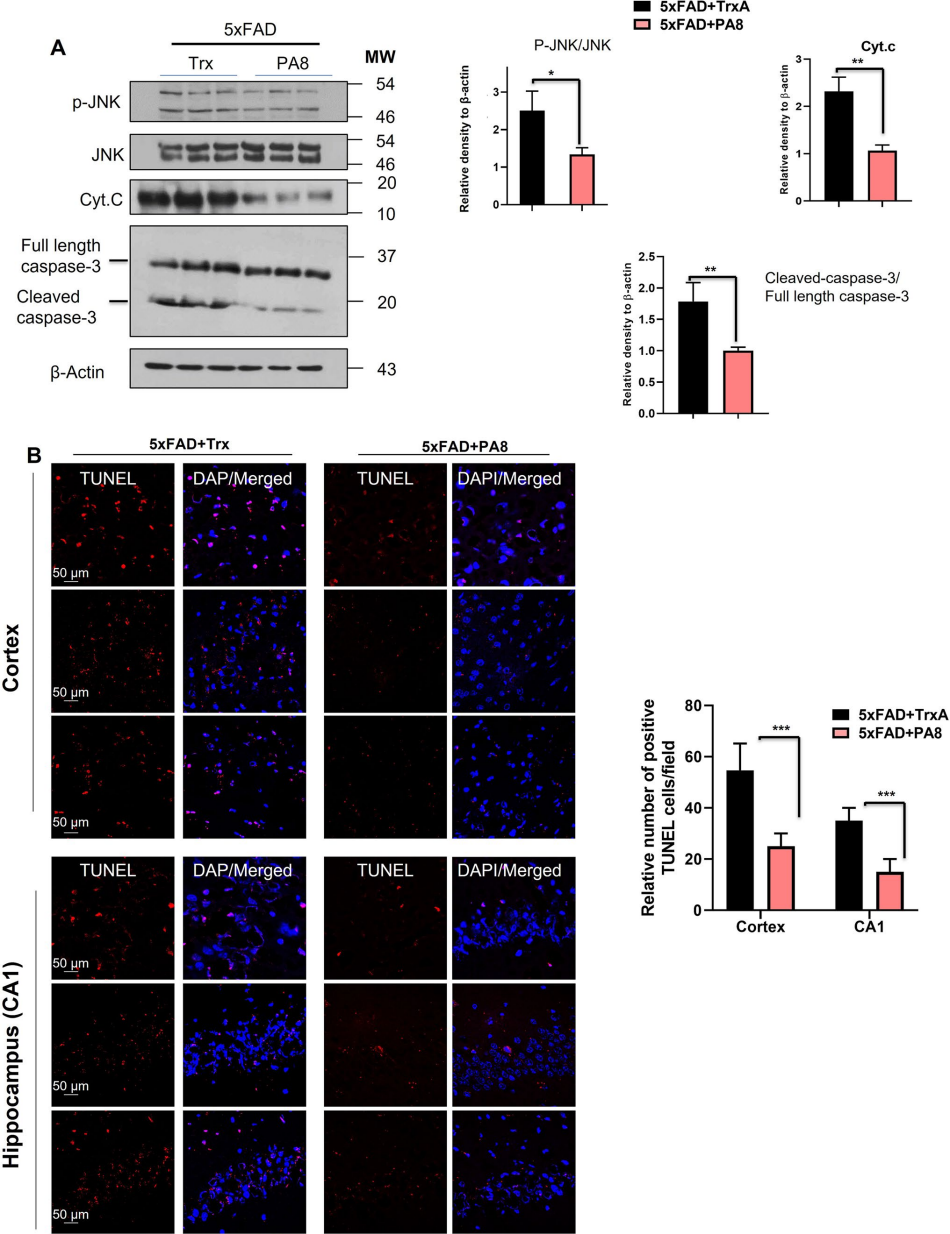
PA8 reduces stress kinase and apoptotic neurodegeneration-related signaling. **A** Immunoblotting and quantification of p-JNK, total JNK, Cyt.C, caspase-3, and β-actin in the three different brain homogenates of individual Trx- and PA8-treated 5XFAD mice. We quantified the ratio of p-JNK with total JNK, cleaved caspase-3 with full length caspase-3 and then normalized with the β-actin. β-Actin was used as a loading control. The data are expressed as the means ± SEM for the representative proteins (*n* = 3 female mice/group) and the number of independent immunoblotting experiments = 3. Significance = *p < 0.05; **p < 0.01. **B** Confocal images of TUNEL-positive cells (red) and DAPI (blue) in the cortex and CA1 region of hippocampus of Trx- and PA8-treated 5XFAD mice. Data are expressed as the means ± SEM for *n* = 3 female mice/group, and the number of independent confocal experiments = 3. Magnification: 63X. Scale bar = 50 μm. Significance = ***p < 0.001

Numerous studies showed that the AβO–PrP interaction instigates various downstream kinases and triggers neurodegeneration through activation of Fyn kinase [reviewed in 19]. Hence, we assessed the effect of PA8 on the stress-activated protein kinase phospho-c-Jun N-terminal Kinase 1 [p-JNK1] (T183/Y185) and its associated mitochondrial apoptotic neurodegenerative signaling. We found significantly lower p-JNK levels in brain homogenates of PA8-treated compared to Trx-treated 5XFAD mice (Fig. 8A; *p < 0.05). It is well-known that activated p-JNK mediates the activation and release of mitochondrial cytochrome C (Cyt.C), which subsequently triggers activation of caspases and apoptotic neurodegeneration. Our immunoblotting results indicate that brain homogenates of PA8-treated 5XFAD mice had significantly lower Cyt.C and cleaved caspase-3 levels as compared to Trx-treated 5XFAD mice (Fig. 8A; **p < 0.01). Additionally, PA8 significantly reduced the number of TUNEL-positive neuronal cells as compared to Trx-treated 5XFAD mice (Fig. 8B). As a control, we stained the sections using the same conditions but without the adding of TUNEL reaction mixture. No staining was detectable (Fig. S8), which confirmed specificity of the signals in Fig. 8B. Collectively, these results confirmed that PA8 rescues the downstream pathologies of the AβO–PrP–Fyn axis, namely neuroinflammation and apoptotic neurodegeneration as observed in AD.

## Discussion

The preventive and therapeutic applications of peptide aptamers have emerged in a wide range of chronic and incurable diseases. Peptide aptamers are superior to conventional and classical protein-based therapeutics (e.g., antibodies, antibody fragments and other non-antibody scaffold-based molecules) because of their small size, convenient designing and low cost, high-yield production. The target-specific peptide moiety is inserted into a scaffold protein, which results in a constrained conformation and higher binding affinity compared to native peptides. In short, peptide aptamers are stable, specific, and non-toxic research tools which have the advantage of effectively binding to their targets with high affinity and specificity. Hence, besides its therapeutic effects, peptide aptamers can be used to identify and validate therapeutic targets [25–32]. Our group previously developed peptide aptamers to target PrP^C^ and interfere with the conversion to PrP^Sc^, its misfolded and infectious isoforms associated with the pathogenesis of prion diseases [20–24]. Herein, our main objectives were to use PA8 for targeting the AβO–PrP–Fyn axis and test for the first time the potential therapeutic and memory improving effects of PA8 treatment in the well-known transgenic 5XFAD mouse model of AD. Our intriguing findings show that PA8 interferes with AβO–PrP interaction and prevented neurotoxicity in cell lines, primary neurons, and the 5XFAD mouse model of AD. Moreover, PA8 treatment improves the memory function of 5XFAD mice and prevents Fyn kinase activation and associated activated gliosis and apoptotic neurodegeneration. Similarly, our findings are supported by other studies that target the AβO–PrP interaction as a valuable therapeutic approach to rescue AD pathologies [12–16].

According to the amyloid cascade hypotheses, mounting studies suggest that along with Aβ plaques, AβO species are the major and neurotoxic species. Both Aβ plaques and AβO species are considered the major pathological characteristics of AD [4–9, 45–48]. It was also shown that soluble AβO species spread among cells and neuropil and, therefore, were considered as a main mediator of synaptic and apoptotic neurodegeneration, which subsequently lead to memory impairment in AD [4–10]. It is widely reported that PrP acts as a high-affinity receptor for AβO and subsequently their interaction triggers neurotoxic effects of AβO. An accumulation and co-localization of PrP^C^ with the diffuse region of Aβ plaques in AD was also reported. Thus, PrP^C^ has a key role in AD pathogenesis [12–15, 49–51]. Nevertheless, the role of PrP^C^ is complicated in neurodegenerative diseases such as AD. AβO were reported to interact with several regions of PrP to mediate their neurotoxic effect [reviewed in 9]. However, most of the studies showed that residues 95–110 of PrP^C^ constitute the main site for AβO interaction [12, 18, 52–58]. Our PA8 binds to aa 100–120, which covers the binding site of AβO [22, 23]. Interestingly, we found that PA8 indeed prevents the interaction of AβO–PrP and significantly reduces the AβO neurotoxicity. Our in vivo results also confirmed that PA8 remarkably reduces the levels of oligomeric forms of Aβ, especially Aβ dodecamer as well as Aβ plaques immunoreactivity (Fig. 3A-C). It also reduced the PrP^C^ signal in immunofluorescence, which might be due to competitive binding of or steric hindrance of anti-PrP antibody binding by either AβO or PA8, with the anti-PrP antibody 4H11 recognizing an N-terminal epitope. This reduction has not been observed by Western blot analysis of PrP^C^ (Fig. S9). Most importantly, our PLA experiment further confirmed that PA8 reduces the AβO–PrP interaction in the cortical and hippocampal regions of the brain in 5XFAD mice as compared to Trx-treated control mice (Fig. 4A, B). The effect of reduced PLA puncta might partially be due to reduced AβO levels, but overall, the reduction of PLA signal in PA8-treated mice (about threefold–fourfold; Fig. 4) appears more pronounced than the AβO reduction (about twofold; Fig. 3), supporting an inhibition of interaction by PA8. It is not clear on the molecular level why Aβ plaques and levels of AβO and specifically dodecamers were reduced in our PA8-treated 5XFAD mice. However, it is possible that in this chronic treatment regimen, the downstream mediators of AβO–PrP interaction have a role in the reduction of Aβ plaque burden and level of Aβ oligomer and dodecamer. Along with that hypothesis, further potential effects of PA8, including activation of non-amyloidogenic and other misfolded protein clearance pathways such as autophagy should be investigated in in vitro and in vivo AD models. Notably, increased Aβ dodecamers were found in aging individuals and patients with mild cognitive impairments [59]. Thus, the beneficial effect of PA8 on reducing AβO, Aβ dodecamer, and Aβ plaques suggests that PA8 would have a potential to rescue both early and late onset of AD.

Fyn kinase is a member of the Src family of non-receptor tyrosine kinases, which is widely expressed throughout the central nervous system and is associated with the AβO–PrP interaction [60]. Seminal studies by Stephen M Strittmatter’s group showed that the AβO–PrP interaction results in the activation of Fyn kinase [12–16, 61]. Additionally, it was found that AβO activate Fyn in dendritic spines in a PrP^C^-dependent manner. Notably, depletion of the prion protein gene (*Prnp*) significantly reduced the elevated level of phosphorylated Fyn and hyperphosphorylation of tau proteins. In line with this, the overexpression of PrP causes an elevation of p-Fyn and tau hyperphosphorylation in both in vitro and in vivo AD models [56]. Our results indicate that PA8 remarkably reduces elevated p-Fyn as compared to Trx-treated 5XFAD mice (Fig. 5A–C). These results were consistent with a study showing that Fyn phosphorylation was inhibited using an antibody to interfere with AβO binding to PrP^C^ [56]. Notably, depletion or inhibition of Fyn alleviates misfolded protein and particularly tau pathologies in tau-overexpressing mice [62, 63]. Further, it was revealed that synthetic compounds which prevent the AβO–PrP interaction are effectively reducing p-Fyn and p-tau in in vitro AD models [64]. These studies suggest that in future, it is required to assess the potential effect of PA8 in tauopathies and other AD models.

Neuroinflammation and neurodegeneration are the most crucial pathogenic features of AD and other neurodegenerative diseases. Recently, it was reported that aberrant Fyn signaling is related to most neurodegenerative diseases namely AD, tauopathies, Parkinson’s disease, multiple sclerosis, brain ischemic stroke, and intracerebral hemorrhage as well as seizures and other brain disorders [65, 66]. Activated Fyn has a pivotal role for proinflammatory mediators and neuroinflammation. Fyn is associated with reactive gliosis and mediates proinflammatory markers such as tumor necrosis factor alpha (TNF-α), interleukin-1beta (IL-1β) and IL-6 as well as the activation of inflammasome (NLRP3). Of note, the reduction, inhibition or depletion of Fyn attenuated proinflammatory markers and neuroinflammation [42, 44, 67]. Likewise, activated Fyn is implicated in triggering mitochondrial apoptotic neurodegeneration and cell death [38, 42, 66, 68]. In our study, we observed less p-Fyn in the PA8-treated 5XFAD mice compared to Trx-treated mice, while there is no change in total Fyn, which indicates that PA8 does not affect basal and total Fyn level, which has a physiological role. Moreover, our results confirm that PA8 reduces p-JNK level as well as the mitochondrial apoptotic neurodegeneration. Collectively, these results indicate that PA8 prevents activated Fyn-associated neuroinflammation and neurodegeneration.

It was reported that PrP^C^ mediates detrimental AβO-induced effects such as a deficit in memory functions and loss of LTP (long-term potentiation) in transgenic mouse models of AD [12, 58, 69]. The chronic administration of anti-PrP^C^ antibody (AZ59) to aged APP/PS1 transgenic mice rescues cognition and synapses in those transgenic AD mice [57]. Further, it was shown that knockout or knockdown of *Prnp* prevented synaptic dysfunction in mice and in vitro AβO studies [51]. Activation of Fyn is one of the mediators for the AβO–PrP-induced synaptic and memory functions. It was also shown that Fyn is localized to the synapses and mediates the AβO–PrP-associated synaptic signaling resulting in deficient memory functions in AD mice [59]. Moreover, inhibition of Fyn activation improves synaptic signaling and memory functions in transgenic and traumatic tauopathy animal model [61]. Interestingly, consistent with these findings for the first time, we describe that our PA8 improves pre-synaptic integrity and the learning and memory functions of 5XFAD mice. Considering these evidences and our results, we attribute the beneficial effects of PA8 in improving synaptic and memory functions of 5XFAD mice to the inhibition of AβO–PrP interaction and its downstream signaling.

Recently, several approaches were used to target PrP–AβO interaction using chemical compounds and anti-PrP nucleic acid (RNA or DNA) aptamers to inhibit PrP^C^–Aβ interaction and Aβ aggregation, which subsequently reduced neurotoxicity [64, 70, 71]. Iida et al. reported that anti-prion protein RNA aptamer R12 disrupts the complex between PrP and AβO oligomer but inhibits the protective effect of PrP and restarting of the Aβ fibrillization [70]. Liu et al. also developed DNA-based aptamers, which dimerized the PrP^C^ (aptamer-induced dimerization (AID) [71]. The AID interferes with AβO − PrP^C^ interaction and produces neuroprotective signaling via shedding of PrP^C^. It prevents AβO-induced mitochondrial dysfunction and apoptosis as measured by the level of activated caspase-3. The AID also reduced proinflammatory cytokines and subsequently ameliorated neuroinflammation [71]. Likewise, Grayson et al. developed synthetic compounds, which have the potential to inhibit the interaction of AβO − PrP^C^ and remarkably reduced the activated p-Fyn and p-tau proteins in in vitro AD models [64]. However, so far, all these studies [64, 70, 71] were performed using in vitro models and did not analyze the in vivo effect of these small compounds, RNA and DNA aptamers. Our findings are promising as for the first time we show that PA8, which targets the PrP–AβO interaction, rescues AD pathologies in an animal model of AD and improves memory functions. Additionally, our results are consistent with the above studies and showed that PA8 reduced the neuroinflammation as well as apoptotic neurodegeneration as downstream effects. However, one limitation of our study is that PA8 was administered via Alzet osmotic pump, which is not feasible for translational and clinical purpose and restricted the use of large numbers of animals. Second, we used 5XFAD mouse models, where PA8 improved the memory functions of these mice. Nevertheless, recently a comprehensive study reported that 5XFAD mice have limitations for translational studies evaluating the potential of therapeutics to treat memory deficits [72] and therefore, in future studies, we plan to address these issues to use other AD mouse models, and to improve the delivery of PA8 through nano-formulation, e.g., encapsulation, nanosphere or liposome preparation of PA8, which can be administered orally or through a parenteral route for long-term treatment of progressive and incurable neurodegenerative diseases. Third, we have only used female mice in our study and we cannot rule out whether the effect of PA8 treatment is sex specific, which will be addressed in future studies; however, women are more affected by AD than men [73]; therefore, PA8 treatment would be relevant for the majority of patients.

In summary, our in vitro and in vivo results demonstrate that PA8 might be valuable as emerging and promising therapeutic tool to prevent and treat AD. Along with the recombinant PA8, our approach has the potential to use PAs as a gene therapy approach in vivo by stable transfection with a transgene encoding secreted versions of the PAs [20]. AD is a complicated and chronic disease with multiple factors and causing mediators. Therefore, it is worth noting that the development of therapeutic tools based on PA8 as recombinant or gene therapy using secreted PA8, which target the PrP^C^–AβO interaction and could reduce not only AβO but also rescued its associated downstream signaling which subsequently prevent neuroinflammation and neurodegeneration, will be advantageous in the successful development of effective therapeutics for AD and other incurable protein misfolding neurodegenerative diseases.

### Supplementary Information

Below is the link to the electronic supplementary material.

Supplementary file1 (DOCX 1965 KB)

## Data Availability

We included all the relevant data in this original manuscript file and/or the supplementary information file. Nevertheless, any additional data related to these findings will be provided to the readers upon reasonable request from the corresponding author.
