# Supplementary material for: Peptide aptamer targeting Aβ–PrP–Fyn axis reduces Alzheimer’s disease pathologies in 5XFAD transgenic mouse model
Source: Cell Mol Life Sci. 2023 May 7;80(6):139. doi: 10.1007/s00018-023-04785-w (PMC10164677; doi:10.1007/s00018-023-04785-w)
Supplement: Supplementary file 1 — Supplementary file1 (DOCX 1965 KB) [file 18_2023_4785_MOESM1_ESM.docx]

***Cellular and Molecular Life Sciences CMLS-D-22-01538-R2***

**Supplementary Material**

**Peptide aptamer targeting Aβ-PrP-Fyn axis reduces Alzheimer’s disease pathologies in 5XFAD transgenic mouse model**

Tahir Ali^1,2,+^, Antonia N. Klein^1,2,+^, Alex Vu^1,2^, Maria I. Arifin^1,2^, Samia Hannaoui^1,2^, Sabine Gilch^1,2*^

^1^ Calgary Prion Research Unit, Department of Comparative Biology & Experimental Medicine, Faculty of Veterinary Medicine, University of Calgary, Calgary, Alberta, Canada.

^2^ Hotchkiss Brain Institute, Cumming School of Medicine, University of Calgary, Calgary, Alberta, Canada.

^+^ Contributed equally

**Running title: Peptide aptamers as novel therapeutics to treat Alzheimer’s disease**

^*^Corresponding author

Sabine Gilch, PhD

Associate Professor

Canada Research Chair in Prion Disease Research

Dept. of Comparative Biology & Experimental Medicine, Faculty of Veterinary Medicine

University of Calgary, 3330 Hospital Drive NW Calgary, AB T2N 4Z6, Canada

Office: HRIC1AC66; Phone: (403) 210-7578

E-mail: [sgilch@ucalgary.ca](mailto:sgilch@ucalgary.ca)


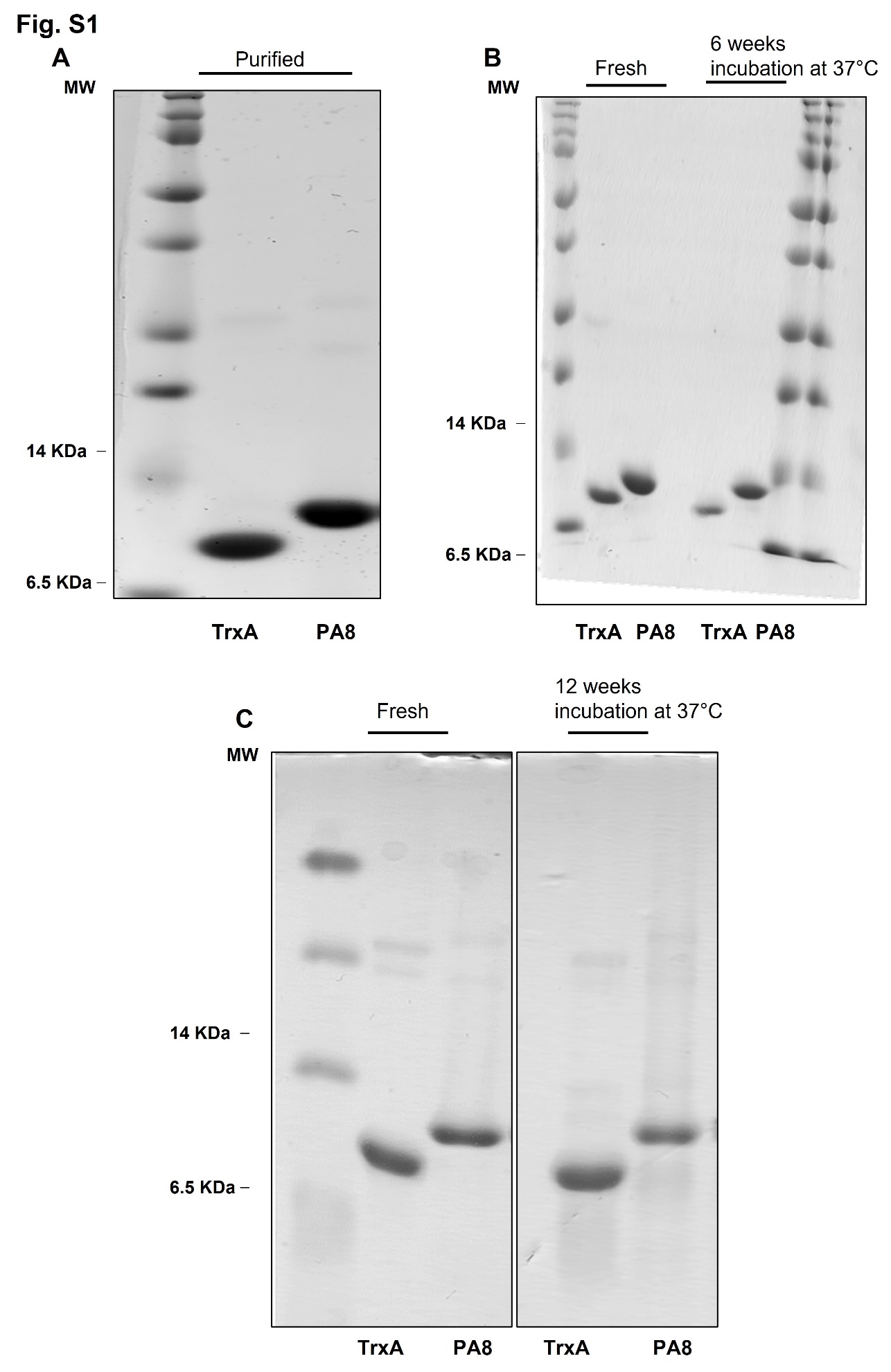


**Fig S1. Confirmation of purity and stability of recombinant PA8.** Two μg of purified PA8 and Trx **(A)** were applied to SDS-PAGE and the gel was stained with Coomassie Blue. Stability was compared for fresh protein or after incubation at 37°C for 6 weeks **(B)** or for freshly purified protein and after 12 weeks of incubation at 37°C **(C)**.


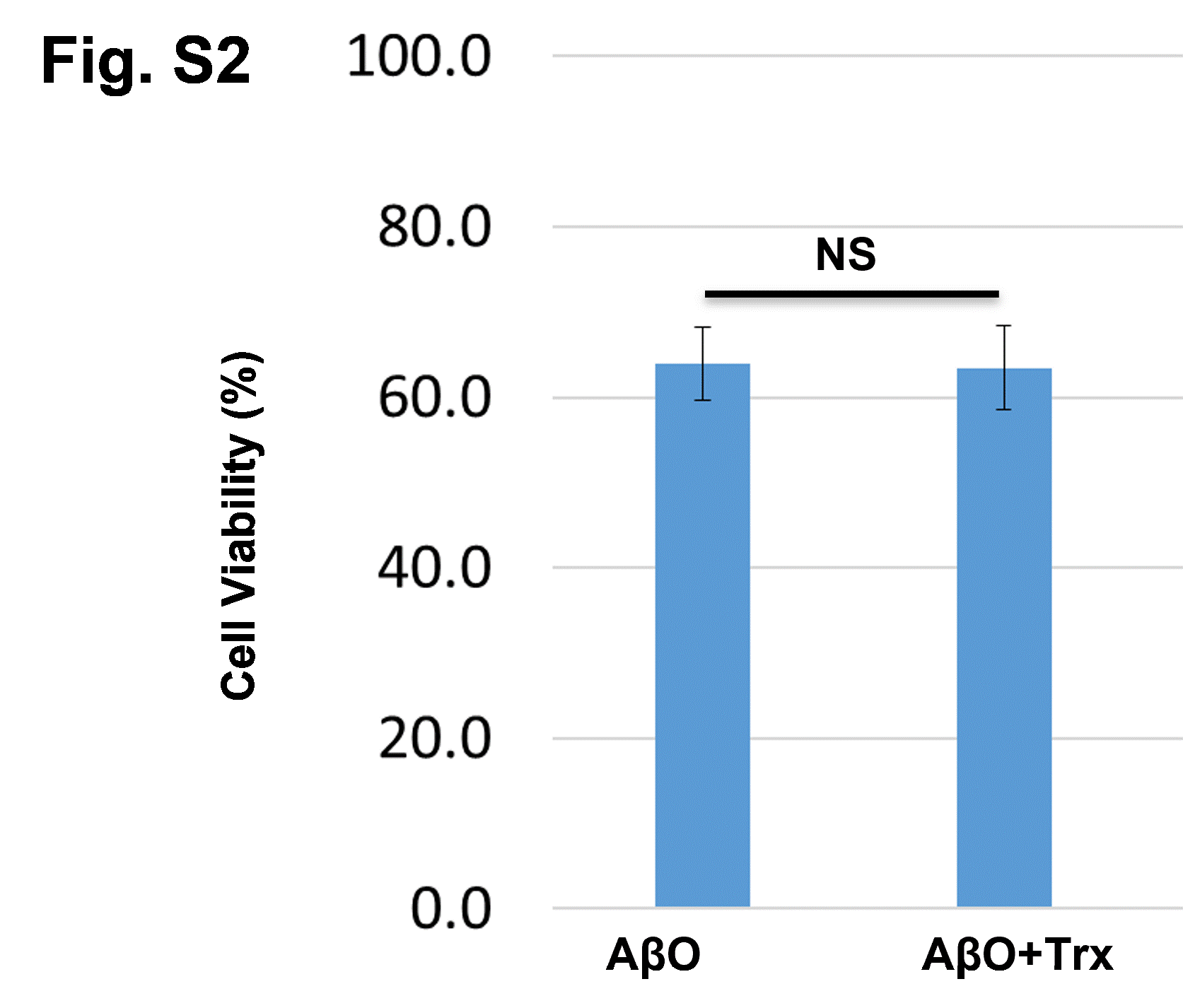


**Fig S2. MTT assay for AβO and AβO+Trx.** N2a cells were treated with AβO (1 µM) alone and AβO 1 µM) and Trx scaffold (10 µg/ml), and after 24 hours MTT assay was performed. The data are expressed as the means ± SEM of three independent MTT experiments = 3. NS= Not significant


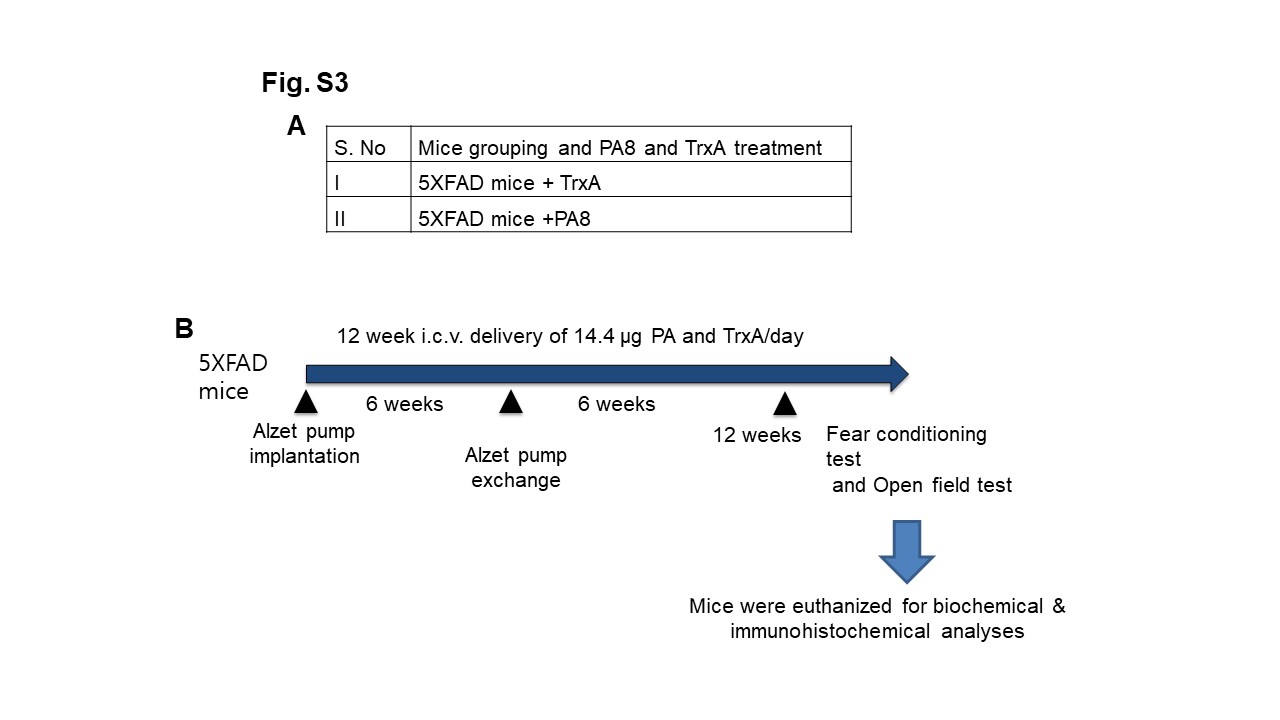


**Fig S3. Schematic diagram of animal bioassay design and treatment paradigm. (A)** Grouping of 5XFAD mice. **(B)** Represents the dosage regimen of PA8 and Trx to female 5XFAD mice and the experimental scheme of the study.


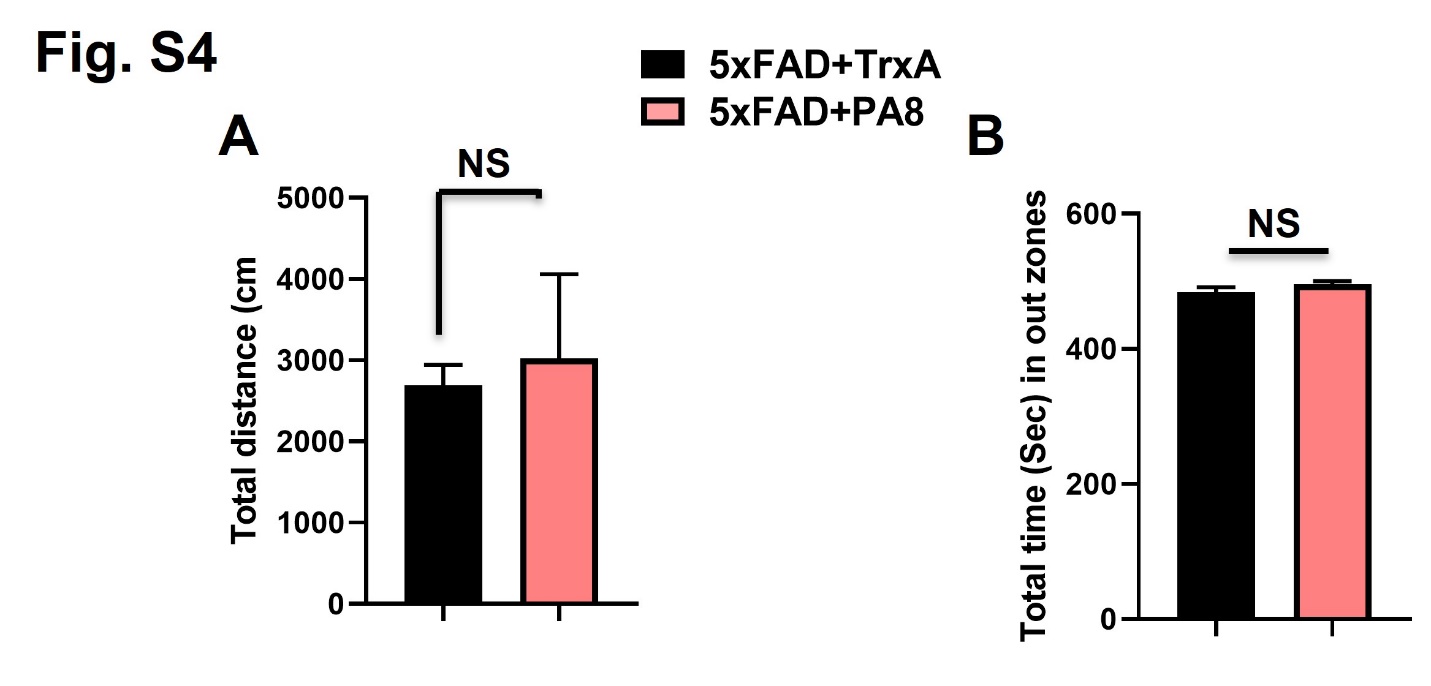


**Fig S4. PA8 and TrxA treatment does not affect the anxiety and motor behavior of 5XFAD mice. (A)** Quantitative analysis of the total distance covered by the mice in the open field box. **(B)** Quantitative analysis of the total time spent in the outer zones of open field box. The histograms show the means ± SEM for the mice (7 female mice/group for PA8 and 5 female mice/group for Trx). No significant difference was observed between Trx and PA8 treated mice.


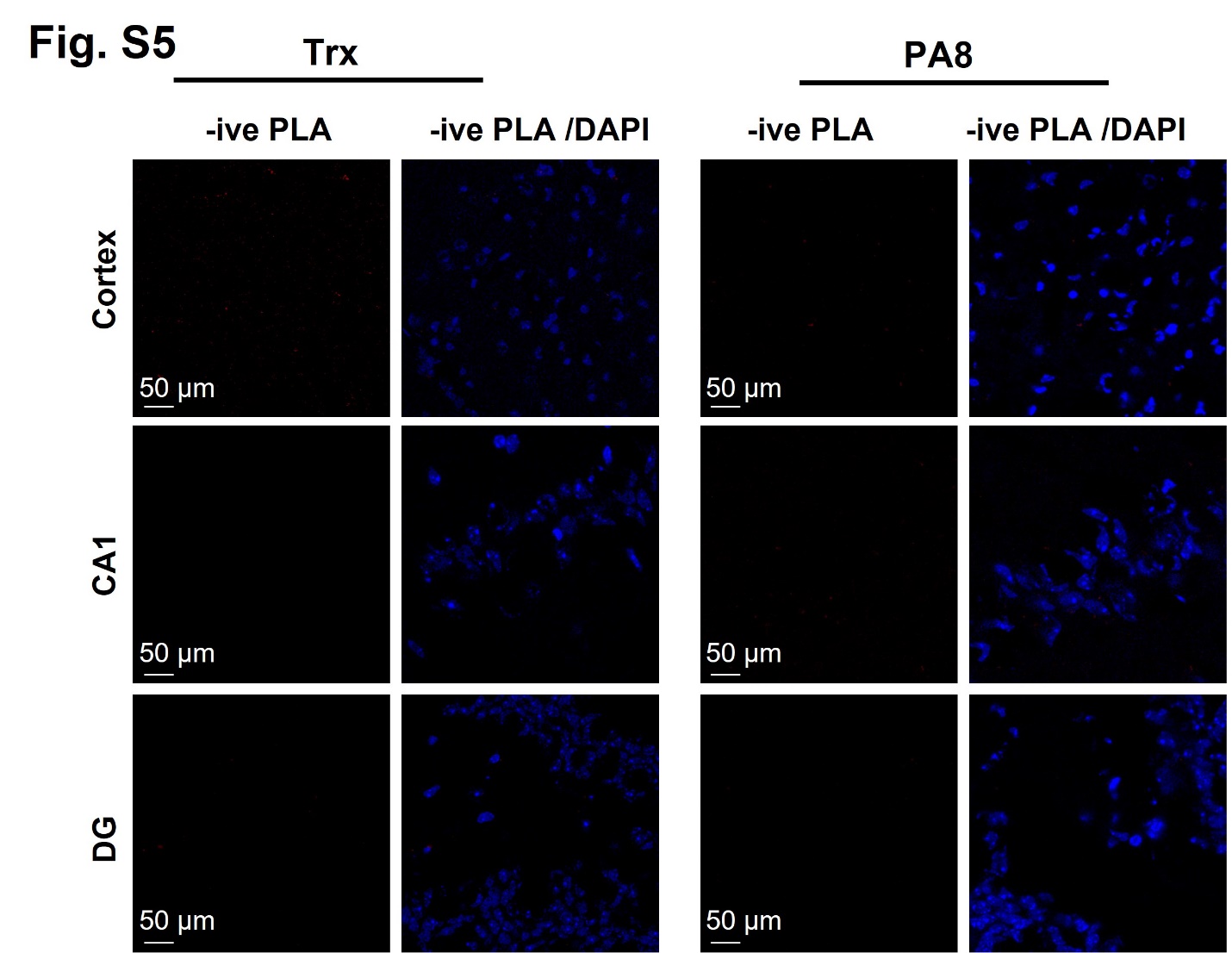


**Fig. S5.** **Negative (-ive) PLA staining.** Representative images of PLA. For the PLA negative control, omitted one of the antibodies for interaction (AβO or PrP). The red color represents the PLA signal while blue is DAPI. We did not observe any PLA positive puncta for PLA with single antibody.


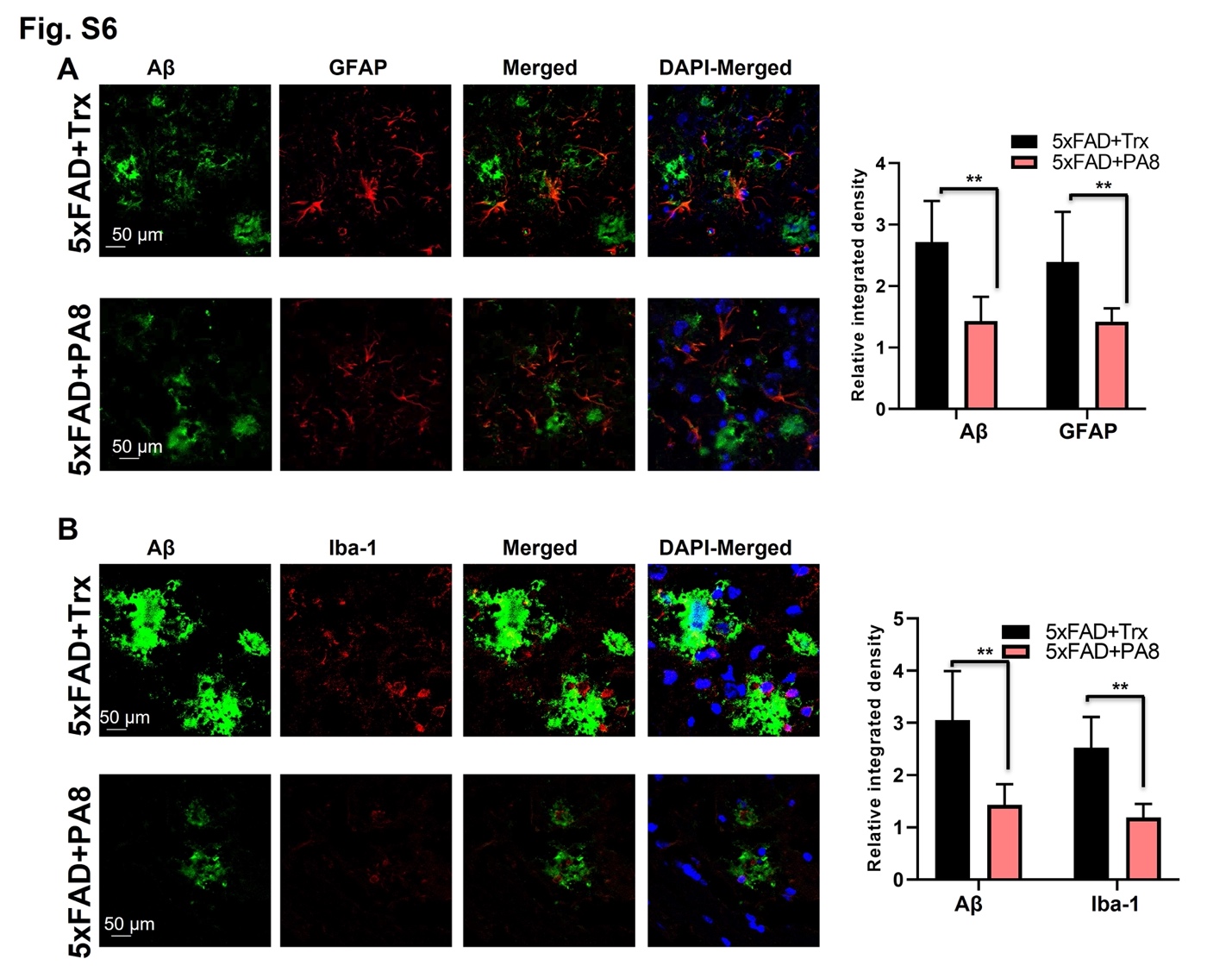


**Fig. S6. Double immunofluorescence of Aβ and activated gliosis in 5XFAD mice. (A)** Confocal images with double immunofluorescence staining of GFAP (red) and Aβ (green) in the brain of Trx- and PA8-treated 5XFAD mice. **(B)** Confocal images of double immunofluorescence of Iba-1 (red) and Aβ (green) in the brain of Trx and PA8 treated 5XFAD mice. Data are expressed as the means ± SEM for n = 3 female mice/group, and the number of independent confocal microscopy experiments = 3. Magnification: 63X. Scale bar = 50 μm. Significance = **p<0.01.


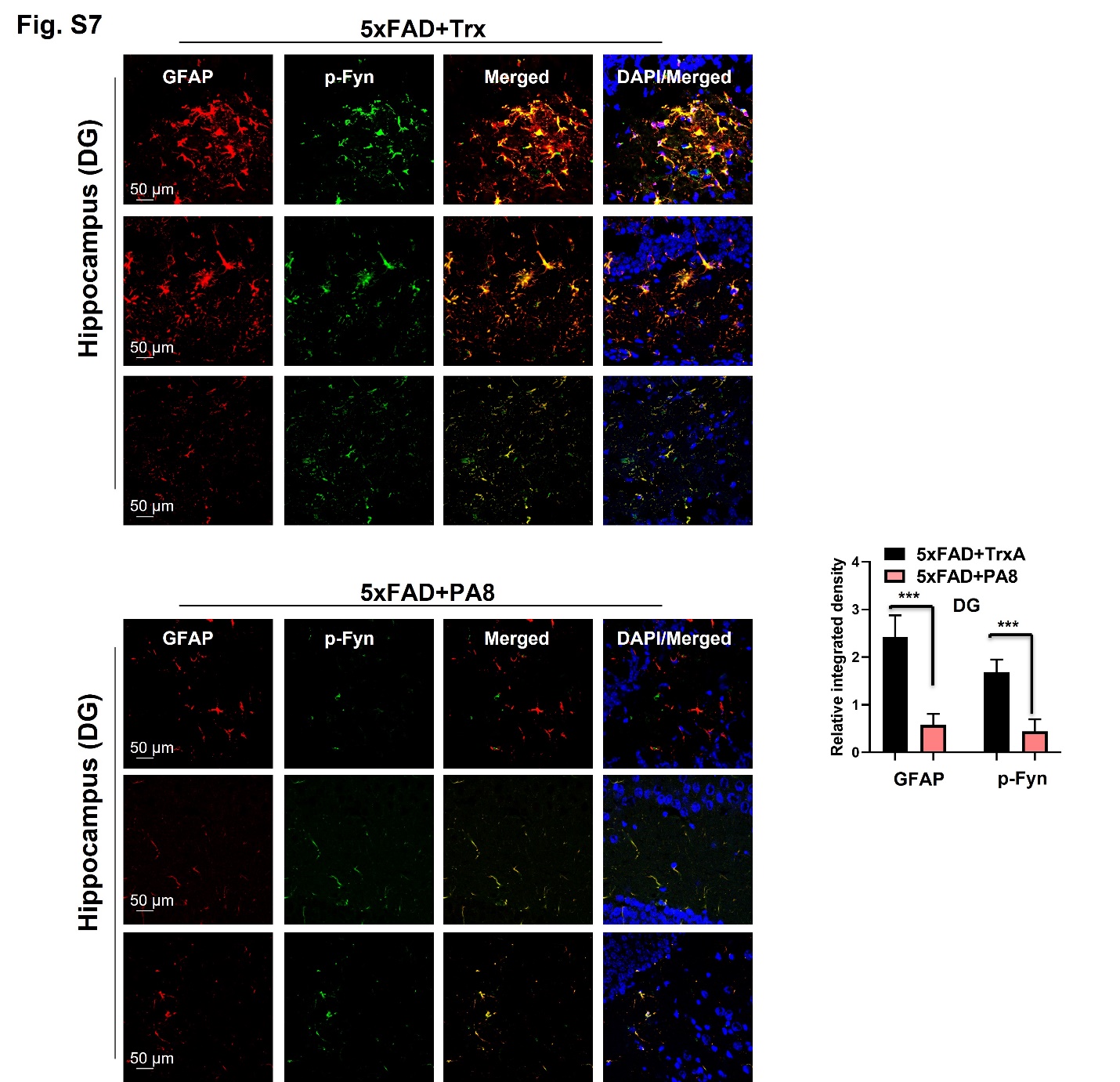


**Fig. S7.** Confocal images of double immunofluorescence of GFAP (red) and p-Fyn (green) in the DG regions of brain of Trx- and PA8-treated 5XFAD mice. Data are expressed as the means ± SEM for n = 3 female mice/group, and the number of independent confocal experiments = 3. Magnification: 63X. Scale bar = 50 μm. Significance = ***p<0.001.


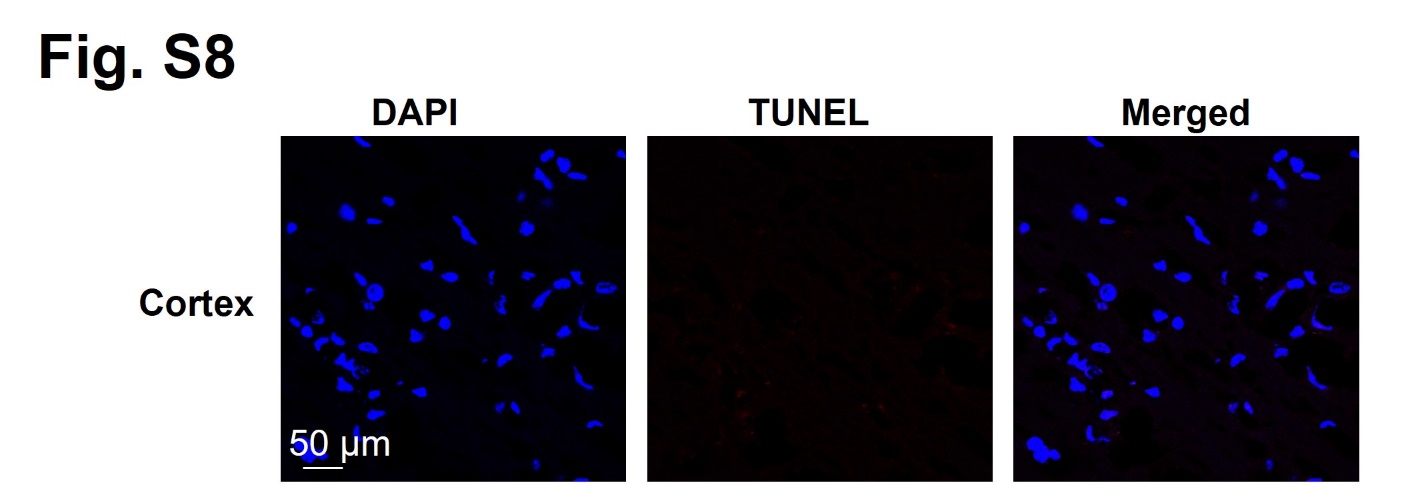


**Fig S8. Negative control for the TUNEL assay.** For negative control, we performed the staining with the same conditions but without adding the TUNEL reaction mixture. The negative controls indicate no TUNEL positive cells. Magnification: 63X. Scale bar = 50 μm.


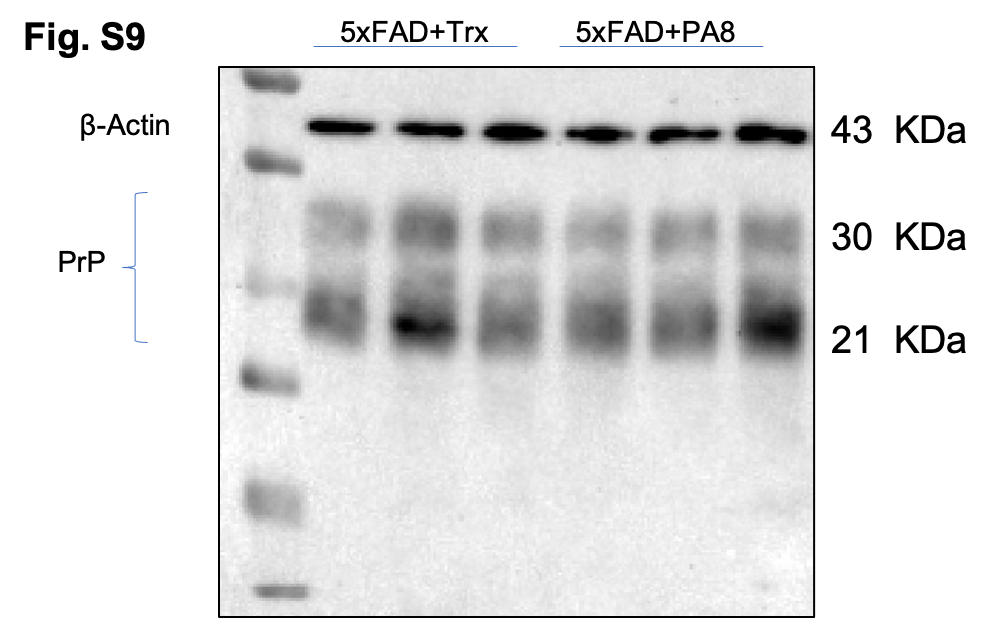


**Fig S9. Effects of PA8 on PrP.** Immunoblotting of of PrP using m4H11 antibody and β-Actin in the three different brain homogenates of individual Trx- and PA8-treated 5XFAD mice.
